# Revisiting Turing’s Chemical Basis of Morphogenesis

**DOI:** 10.1007/s11538-026-01629-z

**Published:** 2026-04-07

**Authors:** John J. Tyson

**Affiliations:** 1 Department of Biological Sciences, Virginia Polytechnic Institute and State University, Blacksburg, VA 24061 USA; 2 Present Address: 783 Spring Lane, Lansdale, PA 19446 USA

**Keywords:** Turing patterns, Dissipative structures, Diffusive instability, Chemical oscillations, Bistability, Traveling waves

## Abstract

In a 1952 paper, Alan Turing showed that spatially distributed chemical reactions evolving in time by local kinetic rate laws and in space by unbiased molecular diffusion can develop a stable, time-independent, spatial pattern from a spatially homogeneous, steady state solution subjected to small, spatially periodic perturbations over a critical range of wavelengths. He proposed this mechanism as a potential chemical basis for biological morphogenesis. Although his proposal was initially ignored and remains controversial, Turing’s idea still plays a major role in any discussion of spontaneous pattern formation in biological and chemical systems. Nevertheless, it is safe to say that few people have carefully studied his 1952 paper, which is notoriously difficult to read. For this reason, I am ‘revisiting’ Turing’s paper to help new investigators to understand and appreciate his remarkable contribution to mathematical biology. Along the way, we shall resolve several ‘peculiarities’ of Turing’s reaction mechanisms and numerical simulations, and place his work in context with later textbook examples. The relation of stationary Turing patterns to time-dependent traveling waves of chemical activity is also described.

**Supplementary Information:**

The online version contains supplementary material available at 10.1007/s11538-026-01629-z.

## Introduction

In 1952 Alan Turing published a ground-breaking paper on the chemical basis of morphogenesis (Turing 1952), in which he proposed that certain types of biochemical reactions, when coupled to molecular diffusion, can generate a stable spatial distribution of chemicals, such as stripes of high and low concentrations on a ring of cells or a dappled pattern on a sheet of cells. Turing developed his theory along three lines. First, he presented a linear stability analysis of the spatially homogeneous, time-independent, steady-state solution of a pair of reaction–diffusion equations (RDEs) on a ring of tissue and derived a set of conditions such that the homogeneous steady state (HSS) is stable with respect to long wavelength (homogeneous) perturbations but unstable with respect to non-uniform perturbations of specific finite wavelengths *λ* (0 < *λ*_min_ < *λ* < *λ*_max_ < ∞). Then he illustrated these principles with a ‘numerical example’ based on a set of hypothetical chemical reactions, for which he computed a spatial pattern of three stripes on a ring of 20 cells. Finally, he suggested that his theory might apply to a few biological examples: the ring-like tentacles of *Hydra* and the leaf whorls of *Asperula*, the ‘dappled’ color patterns of animal coats, and the symmetry-breaking event of gastrulation in embryos. In his last paragraph, Turing concluded:

"It must be admitted that the biological examples which it has been possible to give in the present paper are very limited. This can be ascribed quite simply to the fact that biological phenomena are usually very complicated. Taking this in combination with the relatively elementary mathematics used in this paper one could hardly expect to find that many observed biological phenomena would be covered. It is thought, however, that the imaginary biological systems which have been treated, and the principles which have been discussed, should be of some help in interpreting real biological forms".

Turing’s theory of morphogenesis was largely ignored for many years, for a variety of reasons (Ball 2015; Vittadello, 2021). Although Turing considered his mathematical methods ‘relatively elementary,’ complex numbers, Jacobian matrices (Turing’s ‘marginal reaction rate matrix’), and basis functions for the diffusion operator were hardly standard fare for biologists of his day or (indeed) today. Furthermore, his work was overshadowed by Watson and Crick’s famous 1953 paper on the structure of DNA. Only some 14 years later were Turing’s ideas revived by chemical engineers (Gmitro, 1966) and physical chemists (Edelstein 1970; Prigogine, 1968, 1967).^1^ But, at roughly the same time, Lewis Wolpert (1968, 1969) introduced a rival idea—‘positional information’—for understanding spatial patterns of cellular differentiation; an idea more intuitively appealing to developmental biologists (Marcon, 2012; Sharpe 2019).

In 1972 the tide of opinion of Turing’s ideas began to turn, when Segel & Jackson (1972) presented an elegant and comprehensible mathematical analysis of diffusion-driven instabilities in RDEs and a simple example of pattern formation in a predator–prey system; and, even more significantly, when Gierer and Meinhardt (1972) rediscovered Turing’s theory of pattern formation in reaction–diffusion systems and applied the theory in some detail to development and regeneration of *Hydra* polyps. Since then, Turing’s model of spontaneous pattern formation in biochemical systems has been applied—with successes and failures—to many examples of morphogenesis in biological organisms (Ball 2015; Boissonade, 1995; Krause, 2021; Meinhardt 2012; Murray 1989, 1990; Vittadello, 2021) and of pattern formation in purely chemical reactions in continuous-flow, unstirred reactors (Castets, 1990; Konow, 2021; Lengyel, 1991).

Despite—or maybe because of—his genius, Turing’s paper is notoriously difficult to read, even by experts. His linear stability analysis of the HSS seems unnecessarily complex, his proposed chemical reaction examples have peculiarities (concentrations going negative or to infinity), and his numerical simulations are perplexing because the HSS is stable with respect to small amplitude perturbations of any wavelength 0 < *λ* < ∞. In this contribution to the extensive literature on Turing’s theory, my goal is to provide an entrée for newcomers to Turing’s revolutionary ideas about pattern formation in chemical reaction systems by revisiting his linear stability analysis, chemical examples, and numerical simulations, in order to resolve some of their difficulties and to place Turing’s paper in a more modern context.

This review article is structured as follows: **§2** Turing’s first reaction mechanism is used to illustrate how RDEs are derived from reaction diagrams and cast into dimensionless form. **§3** The conditions for diffusion-driven instability of the HSS of RDEs are derived by Turing’s method of linear stability analysis and compared to later approaches by Segel and Jackson (1972) and Lacalli and Harrison (1979). **§4** Illustrating Turing’s method by his first model, we find that, for his parameter values, the HSS is not subject to diffusion-driven instability; but, nonetheless, Turing patterns develop from large amplitude perturbations of appropriate wavelength. **§5** We briefly describe Turing’s second model and suggest (in the Supplementary Information) two revisions to correct a deficiency of the model. **§6** A generalization of many simple chemical examples proposed in the early, post-Turing literature is introduced. Using one of these examples, we illustrate how Turing patterns extend beyond the domain of diffusion-driven instability. **§7** We explore the relations between Turing patterns, chemical oscillations and wave propagation (time-dependent spatial patterns). **§8** We summarize the significance of Turing’s work and provide references to more recent extensions of his basic idea of spontaneous pattern formation in chemical reaction–diffusion-transport equations.

This review of Turing’s theory (and early extensions) emphasizes, by examples, the sorts of chemical reaction mechanisms that support spontaneous pattern formation by the coupling of reaction and diffusion processes that are themselves spatially unbiased. Turing’s linear stability analysis is applied to each example to determine the conditions for diffusion-driven instability of the HSS. Numerical simulations (using MATLAB) confirm the results of Turing’s linear stability analysis, illustrating the strengths and limitations of the theory. To maintain focus of the main text, many of the technical results are presented in the accompanying Online Supplementary Information.

## Reaction–Diffusion Equations

**Fig. 1:**
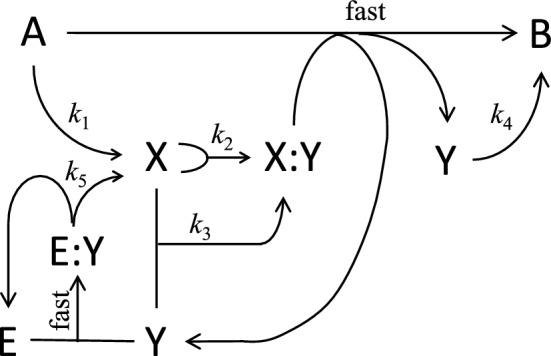
Turing’s Model 1. The overall reaction is A → B, through the intermediates X, Y, X:Y, E and E:Y. The rate at which A is converted into B is limited by the supply of the X:Y complex through the reactions 2X → X:Y and X + Y → X:Y. The enzymatic conversion of Y to X is limited by the concentration of the complex E:Y, where [E:Y] ≈ min([E_T_], [Y])

We begin with Turing’s first model of a chemical reaction system (Table 1 and Fig. 1) that exhibits spontaneous pattern formation. If we assume that the concentration of reactant A is large and constant, the differential equations that describe the reaction and diffusion of the intermediates X and Y are:

1 $$\frac{\partial \widehat{X}}{\partial \widehat{t}}={D}_{x}\frac{{\partial }^{2}\widehat{X}}{{\partial \widehat{s}}^{2}}+{k}_{1}A-2{k}_{2}{\widehat{X}}^{2}-{k}_{3}\widehat{X}\widehat{Y}+{k}_{5}\left[\mathrm{E:Y}\right] ,$$

2 $$\frac{\partial \widehat{Y}}{\partial \widehat{t}}={D}_{y}\frac{{\partial }^{2}\widehat{Y}}{{\partial \widehat{s}}^{2}}+2{k}_{2}{\widehat{X}}^{2}+{k}_{3}\widehat{X}\widehat{Y}-{k}_{4}\widehat{Y}-{k}_{5}\left[\mathrm{E:Y}\right] ,$$

where $\widehat{X}\left(\widehat{s},\widehat{t}\right) \:\mathrm{and} \:\widehat{Y}\left(\widehat{s},\widehat{t}\right)$ are the concentrations (mol/L) of X and Y as functions of space $\widehat{s}$ (cm) and time $\widehat{t}$ (s), and [E:Y] is the concentration of the E:Y complex. Note that [E] + [E:Y] ≡ *E*_T_ = const. Turing assumes, reasonably enough, that formation of the X:Y complex by steps R1.2 and R1.3 is rate-limiting for the fast reaction A + X:Y → B + 2Y. Furthermore, he assumes that [E:Y] = *E*_T_ for all $\widehat{Y}\ge 0$, which is not reasonable because it leads to negative concentrations of Y. Rather, the steady state concentration of the E:Y complex, call it *C*, is given by the real positive solution of

3 $${k}_{1b}\left(\widehat{Y}-C\right)\left(E_{\mathrm{T}}-C\right)=\left({k}_{-1b}+{k}_{5}\right)C,$$

which is

**Table 1:** Turing’s model 1^*^

Ref #	Reaction	Rate law	Rate constants, fixed concentrations
R1.1	A → X	Rate = *k*_1_*A*	*k*_1_ = 6.25 × 10^−8^ s^−1^, *A* = 10^−5^ M
R1.2	2X → X:Y	Rate = *k*_2_*X*^2^	*k*_2_ = 1.094 × 10^4^ M^−1^ s^−1^
R1.3	X + Y → X:Y	Rate = *k*_3_*XY*	*k*_3_ = 1.563 × 10^5^ M^−1^ s^−1^
R1.a	A + X:Y → B + 2Y	Fast	
R1.4	Y → B	Rate = *k*_4_*Y*	*k*_4_ = 6.25 × 10^−5^ s^−1^
R1.b	E + Y → E:Y	Fast	
R1.5	E:Y → E + X	Rate = *k*_5_ [E:Y]	*k*_5_ = 1.719 s^−1^, *E*_T_ = 10^−11^ M

^*^The rate constant values have been computed from Turing’s rate laws, assuming (as he does) that one time unit = 1000 s and one concentration unit = 10^−8^ mol/L. Also, Turing assumes *D*_x_ = 5 × 10^−8^ cm^2^ s^−1^ (typical of a protein diffusing in cytoplasm) and *D*_y_ = 2.5 × 10^−8^ cm^2^ s^−1^

4 $$C=\frac{1}{2}\left[\widehat{Y}+{E}_{\mathrm{T}}+{K}_{\mathrm{M}}-\sqrt{{\left(\widehat{Y}+{E}_{\mathrm{T}}+{K}_{\mathrm{M}}\right)}^{2}-4\widehat{Y}\cdot {E}_{\mathrm{T}}}\right] ,$$

where *K*_M_ = (*k*_−1b_ + *k*_5_)/*k*_1b_ is the Michaelis constant (mol/L) of the enzyme E. Turing assumes that *k*_−1b_ = 0 and *k*_1b_ ≫ *k*_5_, so 0 < *K*_M_ ≪ 1. For our purposes, Eq. (4) is algebraically clumsy, so we shall assume that $C={E}_{T}\cdot \widehat{Y}/({E}_{T}+\widehat{Y})$, which is a good approximation to Eq. (4) when *K*_M_ = *E*_T_/2. In this case, the governing RDEs are

5 $$\frac{\partial \widehat{X}}{\partial \widehat{t}}={D}_{x}\frac{{\partial }^{2}\widehat{X}}{{\partial \widehat{s}}^{2}}+{k}_{1}A-2{k}_{2}{\widehat{X}}^{2}-{k}_{3}\widehat{X}\widehat{Y}+{k}_{5}\frac{{E}_{T}\cdot \widehat{Y}}{{E}_{T}+\widehat{Y}} ,$$

6 $$\frac{\partial \widehat{Y}}{\partial \widehat{t}}={D}_{y}\frac{{\partial }^{2}\widehat{Y}}{{\partial 
\widehat{s}}^{2}}+2{k}_{2}{\widehat{X}}^{2}+{k}_{3}\widehat{X}\widehat{Y}-{k}_{4}\widehat{Y}-{k}_{5}\frac{{E}_{T}\cdot \widehat{Y}}{{E}_{T}+\widehat{Y}} .$$

Our first job is to recast these differential equations in dimensionless form by introducing dimensionless variables:

7 $$X\left(s,t\right)=\frac{\widehat{X}(\widehat{s},\widehat{t})}{{C}_{su}}, Y\left(s,t\right)=\frac{\widehat{Y}(\widehat{s},\widehat{t})}{{C}_{su}}, s=\frac{\widehat{s}}{{S}_{su}}, t=\frac{\widehat{t}}{{T}_{su}},$$

where the ‘standard units’ of concentration, space and time (*C*_su_, *S*_su_ and *T*_su_) are yet to be determined. In terms of the dimensionless variables, Eqs. (5–6) become

8 $$\frac{\partial X}{\partial t}=\frac{{D}_{x}{T}_{su}}{{S}_{su}^{2}}\frac{{\partial }^{2}X}{{\partial s}^{2}}+{k}_{1}{T}_{su}\frac{A}{{C}_{su}}-2{k}_{2}{{T}_{su}{C}_{su}X}^{2}-{k}_{3}{T}_{su}{C}_{su}XY+{k}_{5}{T}_{su}\frac{{E}_{T}}{{C}_{su}}\cdot \frac{Y}{\frac{{E}_{T}}{{C}_{su}}+Y} ,$$

9 $$\frac{\partial Y}{\partial t}=\frac{{D}_{y}{T}_{su}}{{S}_{su}^{2}}\frac{{\partial }^{2}Y}{{\partial s}^{2}}+2{k}_{2}{{T}_{su}{C}_{su}X}^{2}+{k}_{3}{T}_{su}{C}_{su}XY-{k}_{4}{T}_{su}Y-{k}_{5}{T}_{su}\frac{{E}_{T}}{{C}_{su}}\cdot \frac{Y}{\frac{{E}_{T}}{{C}_{su}}+Y} .$$

Following Turing’s lead, we define the standard units as *T*_su_ = 1/*k*_4_ = 1.6 × 10^4^ s = 4.4 h, *S*_su_ = (*D*_x_/*k*_4_)^1/2^ = 0.028 cm = 280 μm, and *C*_su_ = *k*_1_*A*/*k*_4_ = 10^−8^ M = 10 nM, in which case the governing RDEs become

10 $$\frac{\partial X}{\partial t}=\mu \frac{{\partial }^{2}X}{{\partial s}^{2}}+1-{\alpha X}^{2}-\beta XY+\frac{\varphi Y}{\varepsilon +Y}\equiv \mu \frac{{\partial }^{2}X}{{\partial s}^{2}}+F\left(X,Y\right) ,$$

11 $$\frac{\partial Y}{\partial t}=\nu \frac{{\partial }^{2}Y}{{\partial s}^{2}}+\alpha {X}^{2}+\beta XY-\frac{\varphi Y}{\varepsilon +Y}-Y\equiv \nu \frac{{\partial }^{2}Y}{{\partial s}^{2}}+G\left(X,Y\right) ,$$

where

12 $$\begin{aligned}\alpha &= \frac{2{k}_{2}}{{k}_{4}}\cdot \frac{{k}_{1}A}{{k}_{4}}=3.5, \\\beta &= \frac{{k}_{3}}{{k}_{4}}\cdot \frac{{k}_{1}A}{{k}_{4}}=25, \\\varepsilon &= \frac{{k}_{4}{E}_{T}}{{k}_{1}A}=0.001, \\\varphi &= \gamma \varepsilon =\frac{{k}_{5}}{{k}_{4}}\cdot \frac{{k}_{4}{E}_{T}}{{k}_{1}A}=27.5, \\\mu &= 1, \\\nu &= \frac{{D}_{y}}{{D}_{x}}=0.5 .\end{aligned}$$

In these equations, *γ* is the (dimensionless) turnover number of the enzyme, *φ* is its ‘*V*_max_’ and *ε* functions as its Michaelis constant. The steady state solution of Eqs. (10–11) is Y^*^ = 1 and X^*^ the positive root of $\alpha {X}^{2}+\beta X-\varphi /(1+\varepsilon )-1=0$. Notice that Turing placed constraints on his parameter values, 0 < *ε* ≪ 1 and *φ* = *α* + *β* − 1, so that the positive root of the quadratic equation is X^*^ ≈ 1.

**Fig. 2:**
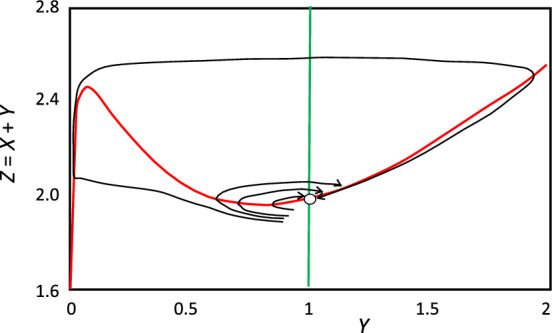
Phase plane portrait (*Z* vs. *Y*, where *Z* = *X* + *Y*) for Turing’s Model 1, Eqs. (10–11). Parameter values *α* = 3.5, *β* = 25, *ε* = 0.01, and *γ* = *α* + *β*–1 = 27.5. The steady state (o) is at Y^*^ = 1, Z* = 1.9915.The green and red curves are the Z and Y nullclines, respectively. Sample trajectories (black curves) indicate that the steady state is globally asymptotically stable, but excitable if *Y* is perturbed below ~ 0.9 (at fixed *X*)

Standard operating procedure, at this point, is to examine the vector field (the ‘phase plane portrait’) of the rate equations in the absence of diffusion, i.e., Eq. (10–11) with *μ* = *ν* = 0. The vector field is easier to visualize if we plot *Z* = *X* + *Y* (rather than *X*) as a function of *Y*; see Fig. 2. All trajectories lead (eventually) to the steady state (Y^*^ = 1, Z^*^ ≈ 2), but perturbations that depress *Y* below ~ 0.9 (at constant *X*) induce a transient drop of *Y* and then a large increase of *Y*, before returning to the asymptotically stable steady state.

## Linear Stability Analysis

Next, we examine the stability of the HSS, (X^*^, Y^*^), with respect to small perturbations: *x*(*s*,*t*) = *X*(*s*,*t*)–X^*^, *y*(*s*,*t*) = *Y*(*s*,*t*)–Y^*^. The nonlinear differential Eqs. (10–11) can be linearized around the HSS to read

13 $$\frac{\partial x}{\partial t}=\mu \frac{{\partial }^{2}x}{{\partial s}^{2}}+ax+by ,$$

14 $$\frac{\partial y}{\partial t}=\nu \frac{{\partial }^{2}y}{{\partial s}^{2}}+cx+dy ,$$

where the constant coefficients (*a*, *b*, *c*, *d*) are the partial derivatives $\left(\frac{\partial F}{\partial X},\frac{\partial F}{\partial Y},\frac{\partial G}{\partial X},\frac{\partial G}{\partial Y}\right)$ evaluated at the steady state X^*^, Y^*^. For example, for Turing’s Model 1: $a=-2\alpha {X}^{*}-\beta {Y}^{*}\approx -\left(2\alpha +\beta \right)=-32, b=-\beta {X}^{*}+\frac{\varepsilon \varphi }{{\left(\varepsilon +{Y}^{*}\right)}^{2}}\approx -\beta =-25, c=-a\approx +32, d=-b-1\approx +24.$

We look for solutions of Eqs. (13–14) of the form

15 $$\left(\begin{array}{c}x(s,t)\\ y(s,t)\end{array}\right)=\left(\begin{array}{c}\xi \\ \eta \end{array}\right){e}^{pt}\mathrm{cos}\left(qs\right) .$$

Non-trivial solutions (${\xi }^{2}+{\eta }^{2}\ne 0$) exist if and only if

16 $${p}^{2}-\left[a+d-\left(\mu +\nu \right){q}^{2}\right]p+\left(ad-bc\right)-\left(a\nu +d\mu \right){q}^{2}+\mu \nu {q}^{4}=0 .$$

In these equations, *q* is the spatial wavenumber of the perturbation (wavelength = *λ* = 2π/*q*) and *p* is the temporal growth rate of the perturbation (the perturbation grows larger with time, i.e., the HSS is unstable, if Re(*p*) > 0). (Note: I am using Turing’s notation, as much as possible.) Eq. (16) is called the ‘dispersion relation’ of the linearized RDEs because, as in optics, it relates the wavelength of a chemical pattern (depending on *q*^*−*1^) to a property of its time-domain (depending on *p*^−1^).

The criteria for diffusion-driven instability are that the HSS is (1) stable with respect to uniform (*q* = 0) perturbations, and (2) unstable with respect to non-uniform perturbations of finite, non-zero wavenumber (0 < *q*_min_ < *q* < *q*_max_ < ∞). The first criterion implies that

17 $$a+d<0~ \mathrm{and}~ ad-bc>0.$$

In this case, $a+d-\left(\mu +\nu \right){q}^{2}<0$, and the second criterion implies that

18 $$H\left({q}^{2}\right)\equiv \left(ad-bc\right)-\left(a\nu +d\mu \right){q}^{2}+\mu \nu {q}^{4}<0.$$

We call this a case of ‘diffusion-driven instability’ because the HSS is ‘kinetically stable’ with respect to uniform perturbations, for which the local concentration changes due to diffusion ($\mu {\partial }^{2}x/\partial {s}^{2}$ and $\nu {\partial }^{2}y/\partial {s}^{2}$) are 0, but ‘diffusively unstable’ because nonuniform perturbations (of wavenumber *q*) induce diffusively driven, local concentration changes that interact with local reaction kinetics to amplify specific nonuniform perturbations.^2^

Because *ad* – *bc* > 0, a necessary condition for diffusion-driven instability of the HSS is

19 $$a\nu +d\mu >0 .$$

Combining the inequalities in (17) and (19), we must insist that *a* and *d* have opposite signs, and that *b* and *c* have opposite signs. Following Turing, we shall assume (without loss of generality) that *a* < 0 and *d* > 0; in which case, 0 < *ν*/*μ* < *d*/|*a*| <  1; i.e., *ν*, the diffusion constant of the ‘self-activating’ variable Y, must be less than *μ*, the diffusion constant of the ‘feedback’ variable X. Furthermore, the Jacobian matrix must have one of two possible sign patterns

20 $$\left[\begin{array}{cc}a& b\\ c& d\end{array}\right]=\left[\begin{array}{cc}-& +\\ -& +\end{array}\right] \:\mathrm{or} \:\left[\begin{array}{cc}-& -\\ +& +\end{array}\right] .$$

These matrices imply that X and Y are involved in a negative feedback loop (*bc* < 0); in the first case Y activates X and X inhibits Y, in the second case X is the activator and Y the inhibitor. Because *a* < 0 and *d* > 0 (by Turing’s convention), species Y is always the self-amplifying variable. For the first sign pattern, the self-amplifying variable is also the ‘activator’ of the negative feedback loop, so this case is called an ‘activator-amplified negative feedback loop’. In this terminology, the second sign pattern is an ‘inhibitor-amplified negative feedback loop’ (Novak, 2008).^3^

Following the methodology (but not the notation) of Segel and Jackson (1972) on p. 549, let *Q* = *q*^2^ and find the minimum of *H*(*Q*) at *Q*_min_ = (*aν* + *dμ*)/2*μν*,

21 $$H\left({Q}_\mathrm{min}\right)=\left(ad-bc\right)-\frac{{\left(a\nu +d\mu \right)}^{2}}{4\mu \nu } .$$

For the HSS to be diffusively unstable, *H*(*Q*_min_) must be a negative number; hence, a stricter requirement for diffusive instability is

22 $$a\nu +d\mu >2\sqrt{\mu \nu (ad-bc)}>0 .$$

This is inequality (15) in Segel & Jackson(1972).

Turing analyzed the dispersion relation a little differently, writing the solution of Eq. (16)

23 $$\begin{aligned}2{p}_{\pm }&=a+d-(\mu +\nu )Q\\ & \quad \pm \sqrt{{\left(a+d-\left(\mu +\nu \right)Q\right)}^{2}-4(ad-bc-\left(a\nu +d\mu \right)Q+\mu \nu {Q}^{2})}\end{aligned}$$

(where Turing’s *U* is my *Q*) and then rearranging the discriminant

24 $$2{p}_{\pm }=a+d-\left(\mu +\nu \right)Q\pm \sqrt{{\left(\left(\mu -\nu \right)Q+d-a\right)}^{2}+4bc}.$$

This is Turing’s Eq. (9.2). Next, he differentiates this function to find that *p*_+_(*Q*) achieves its maximum value when *Q* = *Q*_max_, where

25 $$\left|\left(\mu -\nu \right)Q_{\max}+d-a\right|=\frac{\mu +\nu }{\sqrt{\mu \nu }}\sqrt{\left(-bc\right).}$$

According to Turing’s assumptions, *μ* – *ν* > 0, *d* > 0 and *a* < 0, so

26 $$Q_{\max}=\frac{1}{\mu -\nu }\left[a-d+\frac{\mu +\nu }{\sqrt{\mu \nu }}\sqrt{(-bc)}\right]$$

and

27 $${p}_{+}\left(Q_{\max}\right)=\frac{1}{\mu -\nu }\left[\mu d-\nu a-2\sqrt{\mu \nu (-bc)}\right].$$

**Fig. 3:**
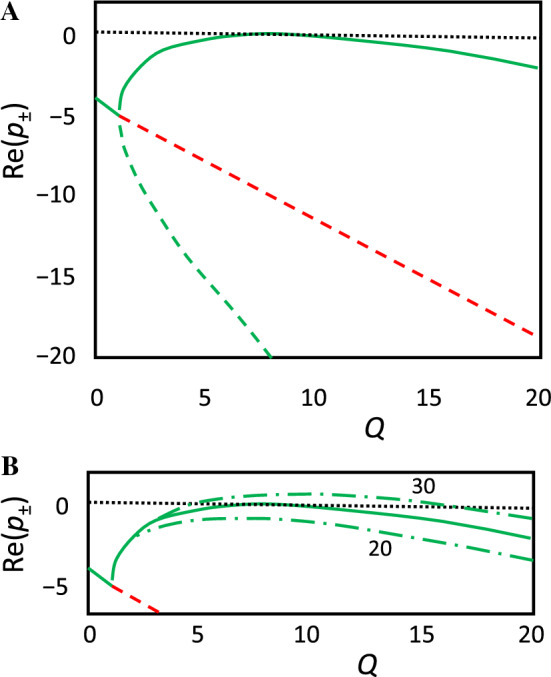
The dispersion relation *p*_±_(*Q*) and Re*p*_±_(*Q*) Eq. (24) for Turing’s Model 1. Solid green curve: *p*_+_(*Q*); and Re*p*_±_(*Q*); dashed green curve: *p*_−_(*Q*); dashed red line: (*a* + *d*–(*μ* + *ν*)*Q*)/2. **A** Turing’s parameter values: *μ* = 1, *ν* = 0.5, *α* = 3.5, *β* = 25 and *φ* = 27.5. Because Re*p*_±_(0) = (*a* + *d*)/2 =  − 4 < 0, the HSS is a stable focus with respect to small uniform perturbations. Because *p*_+_ = 0 for *Q*_max_ = 8, the HSS is marginally stable with respect to small perturbations with wavelength *λ* ≈ 2π/3.16 ≈ 2. **B** The green dash-dot curves are *p*_+_(*Q*) for *β* = 20, *φ* = 22.5 and for *β* = 30, *φ* = 32.5. In the first case, the HSS is stable with respect to small perturbations of any wavelength. In the second case, the HSS is unstable with respect to small perturbations of wavelengths 1.5 < *λ* < 3

These are Turing’s Eq. (9.3). See Fig. 3 for two examples of the dispersion relation, Re*p*_+_(*Q*), for Turing’s Model 1.

For stationary waves of finite wavelength, Turing requires (see his Eqs. (9.4a,b)): *bc* < 0, and, keeping in mind his convention that *a* < 0 < *d*,

28 $$\frac{4\sqrt{\mu \nu }}{\mu +\nu }<\frac{d-a}{\sqrt{-bc}}<\frac{\mu +\nu }{\sqrt{\mu \nu }} ,$$

29 $$\mu d-\nu a>2\sqrt{\mu \nu \left(-bc\right)}>0.$$

The upper bound in Eq. (28) is the condition that *Q*_max_ be real and positive, and Eq. (29) is the condition that *p*_+_(*Q*_max_) > 0, i.e., that the HSS to be unstable for perturbations of wavelength 2π/√*Q*_max_. The lower bound in Eq. (28) is Turing’s condition that $0<\frac{a+d}{2}<{p}_{+}({Q}_\mathrm{max})$, i.e., if the reaction exhibits homogenous limit cycle oscillations (*a* + *d* > 0), the inhomogeneous pattern is expected to predominate if the amplitude of the pattern grows faster than the amplitude of the homogeneous oscillation. Because our definition of diffusion-driven instability of the HSS requires that *a* + *d* < 0 < *p*_+_(*Q*_max_), the lower bound of Turing’s Eq. (9.4a) is redundant for our purposes; but more on this later. Note that condition (29) is equivalent to Segel & Jackson’s condition (22)

The spatial variable in the reaction–diffusion equations can always be scaled so that *μ* = 1. Hence, Eq. (29) can be rearranged to

30 $${a}^{2}{\nu }^{2}-2\left(ad-2bc\right)\nu +{d}^{2}>0 .$$

Because we have already established that 0 < *ν*/*μ* < *d*/|*a*|< 1, inequality (30) implies that

31 $$0<\nu <\frac{d}{\left|a\right|}-\frac{2}{{a}^{2}}\sqrt{\left(ad-bc\right)}\left(\sqrt{\left(-bc\right)}-\sqrt{\left(ad-bc\right)}\right)<\frac{d}{\left|a\right|}<1.$$

This inequality defines the upper limit on the diffusion constant of the self-amplifying variable. In particular, the self-amplifying variable (Y in Turing’s notation) must diffuse more slowly than the feedback variable (X); in some cases, an order of magnitude more slowly. If the RDEs describe a protein regulatory network, then we must consider the fact that all globular proteins of modest molecular weight that diffuse freely in cytoplasm have a diffusion constant ≈ 5 × 10^−8^ cm^2^ s^−1^; so, there must be some exceptional circumstances that slow down considerably the diffusion of the self-amplifying component; for example, it may be polymeric material of high molecular weight, or it may bind reversibly to the relatively immobile cytoskeleton (Korvasova, 2015; Lengyel, 1991). If Y is a protein and X a metabolite, it is easier to imagine that *D*_y_/*D*_x_ < 0.1.

In 1979, Lacalli and Harrison (1979) introduced an elegant graphical representation of Turing’s conditions for diffusion-driven instability of the HSS. By defining

32 $${a}^{\prime}\equiv \frac{-a}{\sqrt{\left(-bc\right)}}>0, { d}^{\prime}\equiv \frac{d}{\sqrt{\left(-bc\right)}}>0 ,$$

they write Turing’s conditions (17a), (17b), (29), (19), and (28) for diffusive instability of the HSS as

33 $$\begin{aligned}\left(\mathrm{i}\right) \ &{d}^{\prime}-{a}^{\prime}<0, \\\left(\mathrm{ii}\right) \ &0<{{a}^{\prime}\cdot d}^{\prime}<1, \\\left(\mathrm{iii}\right) \ &{ d}^{\prime}+{\nu a}^{\prime}>2\sqrt{\nu }, \\\left(\mathrm{iv}\right) \ &{ d}^{\prime}-\nu {a}^{\prime}>0, \\\left(\mathrm{v}\right) \ &{\frac{4\sqrt{\nu } }{1+\nu }<d}^{\prime}+{a}^{\prime}<\frac{1+\nu }{\sqrt{\nu }} .\end{aligned}$$

**Fig. 4:**
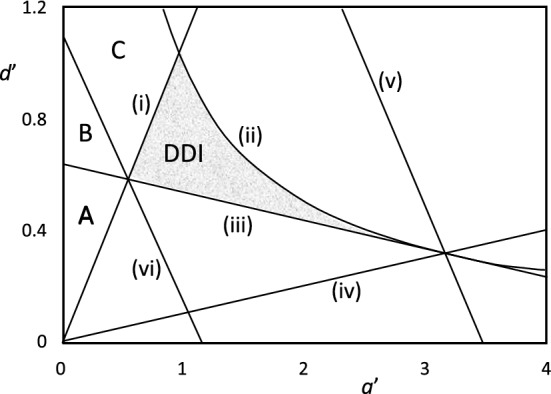
Region of diffusion-driven instability of the HSS in the plane (*a′*, *d′*), where *a′* =  − *a*/√(− *bc*) and *d′* = *d*/√(− *bc*) and (*a*, *b*; *c*, *d*) are the elements of the Jacobian matrix at the HSS. The six lines are (i) *d′* = *a′*, (ii) *d′* = 1/*a′*, (iii) *d′* = 2√*ν*–*νa′*, (iv) *d′* = *νa′*, (v) *d′* = (1 + *ν*)/√*ν*–*a′*, and (vi) *d′* = 4√*ν*/(1 + *ν*)–*a′*, for *ν* = 0.1. The shaded area (DDI) is the region satisfying all of Turing’s conditions (33) for diffusive instability of the HSS. As *ν* increases, this region decreases in area until it disappears at *ν* = 1. Regions A, B and C are explained in the text

In Fig. 4, we illustrate these constraints for the case *ν* = 0.1. The region of diffusive instability is the small shaded region that satisfies all six inequalities (33). We see that the conditions for diffusion-driven instability of the HSS are quite restrictive in the entire space of Jacobian matrices (20).

In regions A, B and C of Fig. 4, *d′* > *a′* and *a′*·*d′* < 1, i.e., *a* + *d* > 0 and *ad*–*bc* > 0, so the HSS is unstable with respect to long-wavelength perturbations, and the RDEs develop homogeneous limit cycle oscillations. In region A, *p*_+_(*Q*_max_) < 0, so Turing patterns are not expected. In region B, 0 < *p*_+_(*Q*_max_) < (*a* + *d*)/2, so we expect the homogenous oscillations to dominate over the Turing pattern. In region C, 0 < (*a* + *d*)/2 < *p*_+_(*Q*_max_), so we expect the Turing pattern to displace the homogenous oscillations. These expectations will be confirmed later by simulations.

## Pattern Formation in Turing’s First Model

On p. 60 of his paper, Turing says apologetically, “The numerous approximations and assumptions that have been made in the foregoing analysis may be rather confusing to many readers. In the present section it is proposed to consider in detail a single example of the case of most interest [stationary waves of finite wavelength].” He then introduces his first model, on p. 61. Unexpectedly, he does not apply his conditions for diffusion-driven instability to the model but embarks abruptly on a lengthy discussion of his numerical calculations, which “were mainly obtained with the aid of the Manchester University Computer.”

Before discussing the numerical simulation, let us apply Turing’s theory to Model 1. As we saw, the HSS is X^* ^≈ Y^*^ = 1, provided $\varphi =\alpha +\beta -1\quad \mathrm{and}\quad  0 < \varepsilon \ll 1$, and the elements of the Jacobian matrix are

34 $$J=\left[\begin{array}{cc}a& b\\ c& d\end{array}\right]\approx \left[\begin{array}{cc}-\left(2\alpha +\beta \right)& -\beta \\ 2\alpha +\beta & \beta -1\end{array}\right] .$$

**Fig. 5:**
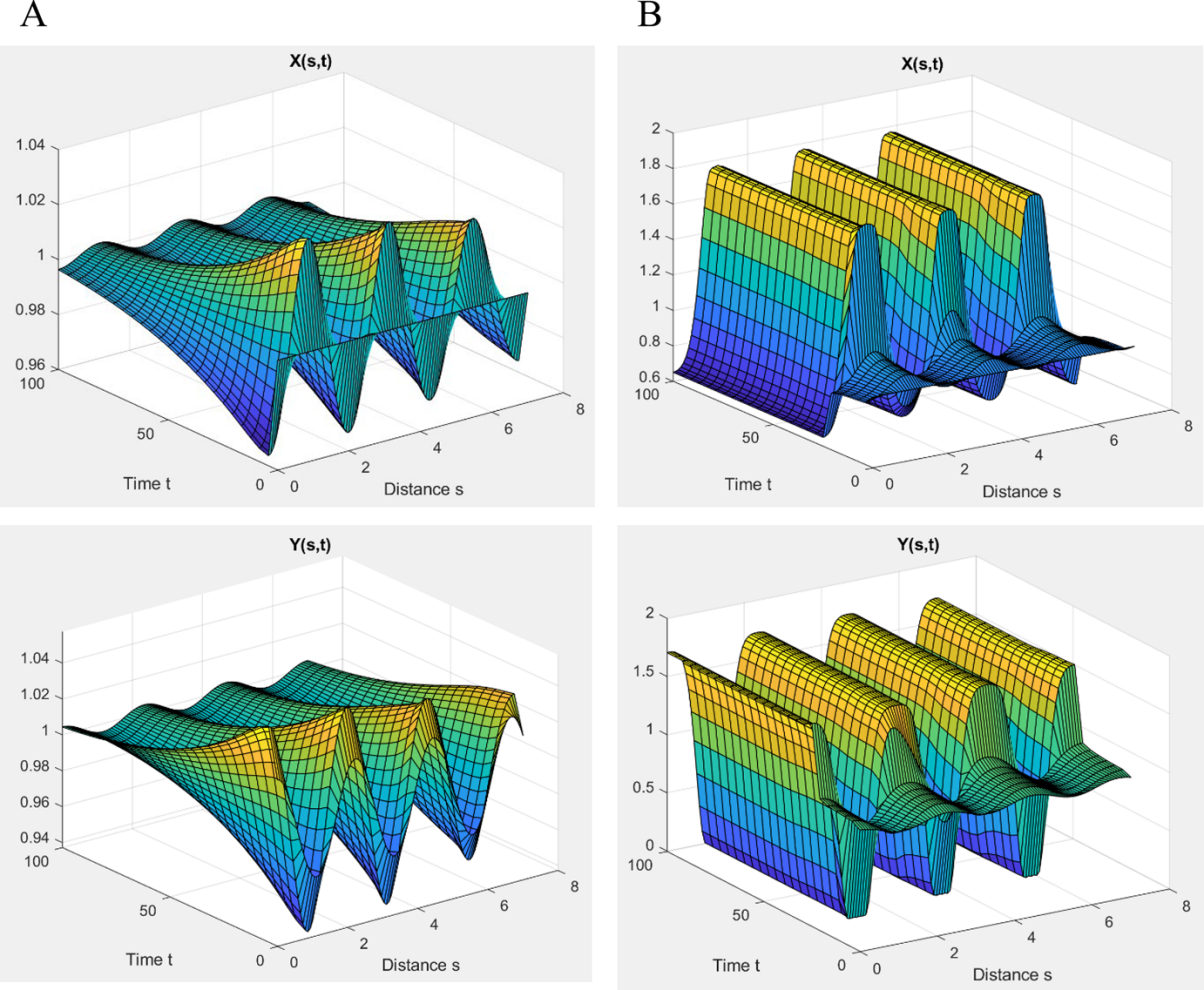
Stable spatial patterns for Turing’s first model, Eqs. (10–11). Parameter values: *α* = 3.5, *β* = 25, *φ* = *α* + *β* – 1 = 27.5, *ε* = 0.001, *μ* = 1, *ν* = 0.5; *L* = 7, Δ*s* = 0.07, predicted *λ* = 2π/√8 = 2.22. **A** Initial conditions: *X*(*s*,0) = 1, *Y*(*s*,0) = 1 + 0.03cos(2π*s*/2.22). Perturbation decays to HSS. **B** In this case, *Y*(*s*,0) = 1 is perturbed by 4%, and, after a significant transient (~ 20 t.u.), the perturbation evolves quickly to a stable pattern with *λ* = 7/3 = 2.33 = 650 μm. These simulations were carried out in MATLAB, using the code in Suppl. Code 1. Turing’s Model 1 is easily simulated in two spatial dimensions with the web-based simulator, VisualPDE (Walker, 2023), using the link https://visualpde.com/sim/?mini=kVvGdOa0

For this matrix, *a* + *d* =  − (2*α* + 1) < 0 and *ad*–*bc* = 2*α* + *β* > 0, so the HSS is stable with respect to long wavelength perturbations. The conditions for the HSS to be unstable with respect to perturbations of finite wavelength are *β* > 1 and $0<\nu <{(\sqrt{\beta }-1)}^{2}/(2\alpha +\beta )$; or 0 < ν < ½ for Turing’s parameter values *α* = 3.5 and *β* = 25. Curiously, Turing assumes that *ν* = *D*_y_/*D*_x_ = ½; hence, the HSS is weakly stable (*p*_+_  = 0 for *Q*_max_ = 8; see Fig. 3A) for small amplitude, non-uniform perturbations with *λ *≈ 2π/√*Q*_max_ (see Fig. 5A, amplitude = 3%). However, in response to a slightly larger perturbation (Fig. 5B, amplitude = 4%), Model 1 develops a robustly stable Turing pattern. This pattern, once established, persists for values of *ν* = *D*_y_/*D*_x_ as large as 0.64 (see Suppl. Fig. S1).^4^

Why did Turing choose his parameter values to lie precisely at the point of bifurcation of spatial patterns? Apparently, Turing wanted to illustrate how patterns might arise spontaneously from a stable HSS by slow change of a parameter value that moves the system across the bifurcation point, in order to evoke a pattern. As “evocator,” Turing chose the concentration, *E*_T_, of the enzyme that catalyzes the conversion of Y into X, i.e., the parameter *φ* in Eqs. (10–11) and the parameter *C* + *C′* ≡ *C*_T_ in Turing’s notation. Turing starts his simulation with *φ *≈ 20, where the HSS is stable to all small perturbations (Fig. 3B), and increases *φ* slowly to ~ 30, where the HSS is maximally unstable to small perturbations of *λ *≈ 2. As the “incipient pattern” starts to develop, Turing then decreases *φ* back to its nominal value of 27.5, allowing the final pattern (Fig. 5B) to emerge. The final pattern has the expected wavelength (*λ* = 7/3 = 2.33 ≈ 2π/√8), which, in my space units, is ~ 650 μm. Turing’s final pattern (his figure 3) is comparable, with a wavelength *λ* = 20/3 = 6.7, which is ~ 670 μm in Turing’s space units.

Turing proposed that such foci of activation on a ring of cells might be responsible for the spacing of tentacles on the medusa of hydrozoans. For example, *Hydra vulgaris* has typically 6 tentacles on a medusa of circumference 3 mm, for a spacing of ~ 500 μm, which is surprisingly close to the wavelength, 660 μm, of the Turing pattern in Fig. 5. The time scale for a hydrozoan to regenerate tentacles is ~ 3 d (72 h), which is surprisingly close to the lag time in Fig. 5B: 15 tu × 4.4 h/tu = 66 h.

In Suppl. Text 1, we revise Turing’s Model 1 to bypass his assumption that $\varphi =\alpha +\beta -1$. In this more general context, the model exhibits limit cycle oscillations and a DDI region bounded on one side by a locus of Hopf bifurcations, in accord with the Lacalli-Harrison diagram in Fig. 4.

## Turing’s Second Model

**Fig. 6:**
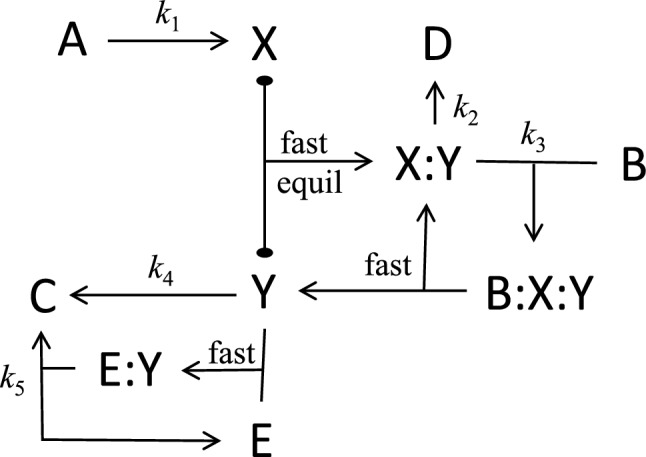
Turing’s Model 2. The overall reaction is A + B → C + D, through the intermediate complexes X:Y, B:X:Y, E and E:Y, where X:Y is an enzyme that converts B to Y, and E is an enzyme that converts Y to C. The X:Y complex is assumed to be in rapid equilibrium with the ‘free’ concentrations of X and Y. The conversion of B to Y is rate-limited by the enzyme–substrate binding reaction (3). The enzymatic conversion of Y to C is limited by the concentration of the enzyme–substrate complex, [E:Y], which is ≈ min([E_T_], [Y])

On p. 65, Turing proposed a second chemical reaction system that he considered less “artificial” to illustrate diffusion-driven instability (see Fig. 6 and Table 2). The RDE for his Model 2 are

**Table 2:** Turing’s model 2^*^

Ref #	Reaction	Rate law	Rate constants, fixed concentrations
R2.1	A → X	Rate = *k*_1_*A*	*k*_1_ = 10^−6^ s^−1^, *A* = 10^−5^ M
R2.a	X + Y → X:Y	Pre-equilibrium	[X:Y] = *X*·*Y*/*K*_d_, *K*_d_ = 10^−5^ M
R2.2	X:Y → D	Rate = *k*_2_*X·Y*/*K*_d_	*k*_2_ = 0.0625 s^−1^
R2.3	B + X:Y → B:X:Y	Rate = *k*_3_*B·X·Y*/*K*_d_	*k*_3_ = 1.25 × 10^4^ M^−1^ s^−1^, *B* = 10^−5^ M
R2.b	B:X:Y → Y + X:Y	Fast	
R2.4	Y → C	Rate = *k*_4_*Y*	*k*_4_ = 6.25 × 10^−5^ s^−1^
R2.c	E + Y → E:Y	Fast	*E*_T_ = 1.2 × 10^−10^ M
R2.5	E:Y → E + C	Rate = *k*_5_[E:Y]	*k*_5_ = 0.0625 s^−1^

^*^The rate constant values have been computed from Turing’s rate laws, assuming (as he does) that one time unit = 1000 s and one concentration unit = 10^−8^ mol/L. Also, Turing assumes, in this case, that *D*_x_ = 2.5 × 10^−8^ cm^2^ s^−1^ and *D*_y_ = 0.625 × 10^−8^ cm^2^ s^−1^

35 $$\frac{\partial \widehat{X}}{\partial \widehat{t}}={D}_{x}\frac{{\partial }^{2}\widehat{X}}{{\partial \widehat{s}}^{2}}+{k}_{1}A-{k}_{2}\frac{\widehat{X}\cdot \widehat{Y}}{{K}_{d}}$$

36 $$\frac{\partial \widehat{Y}}{\partial \widehat{t}}={D}_{y}\frac{{\partial }^{2}\widehat{Y}}{{\partial \widehat{s}}^{2}}-{k}_{2}\frac{\widehat{X}\cdot \widehat{Y}}{{K}_{d}}+{k}_{3}B\frac{\widehat{X}\cdot \widehat{Y}}{{K}_{d}}-{k}_{4}\widehat{Y}-{k}_{5}\frac{{E}_{T}\widehat{Y}}{{E}_{T}+\widehat{Y}}$$

In writing these equations, we are assuming, as Turing does, that the X:Y complex is in rapid pre-equilibrium (step R2.a) to the subsequent reactions 2 and 3, i.e., [X:Y] = [X]·[Y]/*K*_d_, which is a good approximation provided the dissociation constant, *K*_d_ = 10^−5^ M, is much greater than the total concentrations of X and Y, which, as we shall see, are of order 10^−8^ to 10^−7^ M. The kinetics of the enzymatic conversion of Y to C is treated in Model 2 the same as the conversion of Y to X in Model 1.

Using the scaling factors in Suppl. Table 1, we cast Eqs. (35–36) into dimensionless form

37 $$\frac{\partial X}{\partial t}=\mu \frac{{\partial }^{2}X}{{\partial s}^{2}}+\alpha -XY ,$$

38 $$\frac{\partial Y}{\partial t}=\nu \frac{{\partial }^{2}Y}{{\partial s}^{2}}+\left(\beta -1\right)XY-\frac{\varphi Y}{\varepsilon +Y}-Y ,$$

where

39 $$\begin{aligned}\alpha &= \frac{{k}_{1}A/{k}_{4}}{{{k}_{4}K}_{d}/{k}_{2}} = 16, \\\beta &= \frac{{k}_{3}B}{{k}_{2}} = 2, \\\varphi &= \frac{{k}_{5}}{{k}_{4}}\cdot \frac{{k}_{2}{E}_{T}}{{k}_{4}{K}_{d}} = 12, \\\varepsilon &= \frac{{k}_{2}{E}_{T}}{{k}_{4}{K}_{d}} = 0.012, \\\mu &= 1, \\\nu &= \frac{{D}_{y}}{{D}_{x}} = 0.25 .\end{aligned}$$

In line with Turing’s assumed parameter values, we require in general that *β* > 1 and 0 < *ε* ≪ *α*(*β*–1)–*φ*, in which case the kinetic steady state is Y^*^ ≈ *α*(*β*–1)–*φ* > 0 and X^*^ = *α*/Y^*^ ≈ (*β*–1–*φ*/*α*)^−1^.

In this scaling of the PDEs, the Jacobian matrix is

40 $$J=\left[\begin{array}{cc}a& b\\ c& d\end{array}\right] \approx \left[\begin{array}{cc}-[\alpha \left(\beta -1\right)-\varphi ]& -\alpha /[\alpha (\beta -1)-\varphi ]\\ \left(\beta -1\right)[\alpha (\beta -1)-\varphi ]&\varphi/[\alpha(\beta-1)-\varphi]\end{array}\right].$$

Note that *a* < 0, *d* > 0 and $ad-bc=\alpha \left(\beta -1\right)-\varphi >0$. The other requirement for the HSS to be stable with respect to long wavelength perturbations is

41 $$a+d=\frac{\varphi -{[\alpha \left(\beta -1\right)-\varphi ]}^{2}}{\alpha \left(\beta -1\right)-\varphi }<0,$$

which is satisfied if $0<\varphi <\alpha \left(\beta -1\right)-\frac{1}{2}\left(\sqrt{1+\alpha \left(\beta -1\right)}-1\right)<\alpha (\beta -1)$. For Turing’s choice of parameter values (*α* = 16, *β* = 2), this condition implies that 0 < *φ* < 12.47. On p. 65, Turing chooses *φ* = 12 (our *φ* is Turing’s *β*). Because *μ* = 1 in our scaling, Turing’s condition (29) for the HSS to be unstable with respect to perturbations of finite wavelength is

42 $$\frac{\varphi}{\alpha(\beta -1)-\varphi }+\nu \left[\alpha \left(\beta -1\right)-\varphi \right]>2\sqrt{\nu \alpha \left(\beta -1\right)} .$$

For Turing’s parameter values, Eq. (39), inequality (41) is satisfied (just barely), the Jacobian matrix is $$\left[\begin{array}{cc}a& b\\ c& d\end{array}\right]=\left[\begin{array}{cc}-4& -4\\ 4& 3\end{array}\right]$$, and the condition (42) for a diffusive instability is $3+4\nu >8\sqrt{\nu }$, or 0 < *ν* < ¼. Because Turing assumes that *ν* = *D*_y_/*D*_x_ = ¼, the HSS is marginally stable (*p*_+_  = 0) for *Q*_max_ = 4. As for Model 1, the HSS is stable with respect to small perturbations of wavelength = 2π/√*Q*_max _≈ 3 but unstable with respect to large perturbations (see Suppl. Fig. S2). The pattern wavelength, in my space units, is $3\sqrt{{D}_\mathrm{x}/{k}_{4}}$ = 600 μm. Turing reports (his Table 2) a wavelength of 5 cells, or 500 μm.^5^

Turing’s second model has a major problem in that many trajectories ‘blow up,’ i.e., *Y*(*t*) → 0 and *X*(*t*) → ∞ (Erneux 2018). In Suppl. Text S2, we explore two different fixes to this problem.

## Model 3: The Brusselator and Related Models

In the late 1960’s and early 70’s, several authors published models of chemical oscillations and pattern formation that were all based on a common mechanism of self-amplification: X + 2Y → 3Y, *dY*/*dt* =  + *kXY*^2^. The earliest were the ‘Brusselator’ model of Prigogine and Lefever (1968) and a model of glycolysis by Selkov (1968). Later came Gierer and Meinhardt’s (1972) ‘simple depletion model’ of pattern formation and Tyson and Kauffman’s (1975) model of mitotic oscillations in an acellular slime mold. Lefever (1968) published the first study of Turing patterns in the Brusselator, and Lacalli and Harrison (1979) computed Turing patterns in the Tyson-Kauffman model. A later Brusselator-type model by Schnakenberg (Schnakenberg 1979) became the centerpiece of the chapter on Turing patterns in Murray’s (1989) textbook on Mathematical Biology. All of these models are examples of inhibitor-amplified negative feedback loops, Eq. (20).

**Fig. 7:**
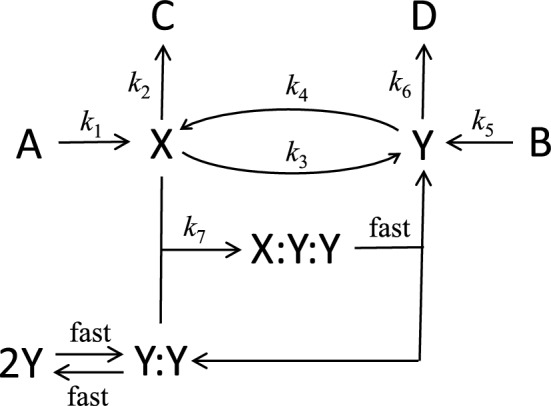
Model 3, after Champneys et al (2021) and Al Saadi et al (2021). The dimerization of Y is assumed to be in pre-equilibrium to step R3.7, so [Y:Y] =  [Y]^2^/*Kd*, provided *K*_d_ ≫ [Y_T_] = [Y] + [Y:Y]

Recently, Champneys et al (2021) and Al Saadi et al (2021) pointed out that all these models are simple versions of the generic model in Fig. 7 and Table 3. Provided $\widehat{Y}\left(\widehat{t}\right)\ll {K}_{d}$, the reaction–diffusion equations for Model 3 are

**Table 3:** Model 3, after Champneys et al (2021) and Al Saadi et al (2021)^*^

Ref #	Reaction	Rate law	Rate constants, fixed concentrations
R3.1	A → X	Rate = *k*_1_*A*	*k*_1_ = 3 × 10^−8^ s^−1^, *A* = 10^−5^ M
R3.2	X → C	Rate = *k*_2_*X*	*k*_2_ = 6 × 10^−6^ s^−1^
R3.3	X → Y	Rate = *k*_3_*X*	*k*_3_ = 6 × 10^−6^ s^−1^
R3.4	Y → X	Rate = *k*_4_*Y*	*k*_4_ = 6 × 10^−6^ s^−1^
R3.5	B → Y	Rate = *k*_5_*B*	*k*_5_ = 6 × 10^−9^ s^−1^, *B* = 10^−5^ M
R3.6	Y → D	Rate = *k*_6_*Y*	*k*_6_ = 6 × 10^−5^ s^−1^
R3.7	X + Y:Y → Y + Y:Y	Rate = *k*_7_*X·Y*^2^/*K*_d_	*k*_7_ = 6 × 10^5^ M^−1^ s^−1^
R3.8	2Y → Y:Y	Pre-equilibrium	[Y:Y] = *Y*^2^/*K*_d_, *K*_d_ = 10^−6^ M

^*^The rate constants have been assigned values in line with Turing’s assumptions. Also, we shall assume, in this case, that *D*_x_ = 5 × 10^−8^ cm^2^ s^−1^ and *D*_y_ = 1.5 × 10^−9^ cm^2^ s^−1^

43 $$\frac{\partial \widehat{X}}{\partial \widehat{t}}={D}_{x}\frac{{\partial }^{2}\widehat{X}}{{\partial \widehat{s}}^{2}}+{k}_{1}A-{k}_{2}\widehat{X}-{k}_{3}\widehat{X}+{k}_{4}\widehat{Y}-\frac{{k}_{7}}{{K}_{d}}\widehat{X}{\widehat{Y}}^{2} ,$$

44 $$\frac{\partial \widehat{Y}}{\partial \widehat{t}}={D}_{y}\frac{{\partial }^{2}\widehat{Y}}{{\partial \widehat{s}}^{2}}+{k}_{3}\widehat{X}-{k}_{4}\widehat{Y}+{k}_{5}B-{k}_{6}\widehat{Y}+\frac{{k}_{7}}{{K}_{d}}\widehat{X}{\widehat{Y}}^{2} .$$

Using the scaling factors in Suppl. Table 1, we cast Model 3 into dimensionless form

45 $$\frac{\partial X}{\partial t}=\mu \frac{{\partial }^{2}X}{{\partial s}^{2}}+\alpha -\left(\gamma +\sigma \right)X+\delta Y-X{Y}^{2} ,$$

46 $$\frac{\partial Y}{\partial t}=\nu \frac{{\partial }^{2}Y}{{\partial s}^{2}}+\beta +\sigma X-\left(\delta +1\right)Y+X{Y}^{2} ,$$

where

47 $$\begin{aligned}\alpha &= \sqrt{\frac{{k}_{7}}{{k}_{6}{K}_{d}}}\cdot \frac{{k}_{1}A}{{k}_{6}} = 0.5,\\\beta &= \sqrt{\frac{{k}_{7}}{{k}_{6}{K}_{d}}}\cdot \frac{{k}_{5}B}{{k}_{6}} = 0.1,\\\gamma &= \frac{{k}_{2}}{{k}_{6}} = 0.1,\\\delta &= \frac{{k}_{4}}{{k}_{6}} = 0.1,\\\sigma &= \frac{{k}_{3}}{{k}_{6}} = 0.1,\\\mu &= 1,\\\nu &= \frac{{D}_{\mathrm{y}}}{{D}_{\mathrm{x}}} = 0.03 \end{aligned}$$

The condition, $\widehat{Y}\ll {K}_{d}$, becomes $Y\ll \sqrt{\frac{{k}_{7}{K}_{d}}{{k}_{6}}}=100$. Table 4 correlates the parameters in Eq. (47) with the free parameters in the simplified models common in the literature.

**Table 4:** Parameter settings for reduced versions of Model 3

Model 3	Champneys Al Saadi	Prigogine Lefever	Selkov	Gierer Meinhardt	Tyson Kauffman	Schnakenberg
*α*	*b*	0	*α*	*α*	*a*	*b*
*β*	*a*	*A*	0	*β*	0	*a*
*γ*	*j* − *d*	0	0	*γ*	0	0
*δ*	*h*	*B*	0	0	0	0
*σ*	*d*	0	0	0	*b*	0
1	*c* − *h*	1	1	1	1	1

^*^All models contain reactions R3.6 and R3.7 and are scaled so that *k*_6_ → 1 and *k*_7_/*K*_d_ → 1

The Al Saadi-Champneys model can be simulated with VisualPDE (Walker, 2023) in one- or two spatial dimensions, using the links https://visualpde.com/sim/?mini=1TtZ7vBy and https://visualpde.com/sim/?mini=zzHCFV_j, respectively.

Model 3 is rather clumsy to analyze in full generality, so let us focus on the Tyson Kauffman model, with two parameters, *α* and *σ*. The unique HSS of this model is Y^*^ = *α* and X^*^ = *α*/(*σ* + *α*^2^), and the Jacobian matrix for this model is

48 $$J=\left[\begin{array}{cc}a& b\\ c& d\end{array}\right]=\left[\begin{array}{cc}-({\alpha }^{2}+\sigma )& -\frac{2{\alpha }^{2}}{{\alpha }^{2}+\sigma }\\ {\alpha }^{2}+\sigma & \frac{{\alpha }^{2}-\sigma }{{\alpha }^{2}+\sigma }\end{array}\right] .$$

The determinant and trace of the Jacobian matrix are

49 $$ad-bc={\alpha }^{2}+\sigma >0,\: \mathrm{and}\: a+d=-\frac{{\left({\alpha }^{2}+\sigma \right)}^{2}-{\alpha }^{2}+\sigma }{{\alpha }^{2}+\sigma }.$$

Turing’s conditions (17) for diffusion-driven instability of the HSS are satisfied provided *d* > 0, i.e., 0 < *σ* < *α*^2^; and *a* + *d* < 0, i.e.,0 < *α* < 1 and,

50 $${\sigma }^{2}+\left(1+2{\alpha }^{2}\right)\sigma -{\alpha }^{2}\left(1-{\alpha }^{2}\right)>0\text{ or }0<\sigma <\frac{1}{2}\left(\sqrt{1+8{\alpha }^{2}}-1-2{\alpha }^{2}\right)$$

**Fig. 8:**
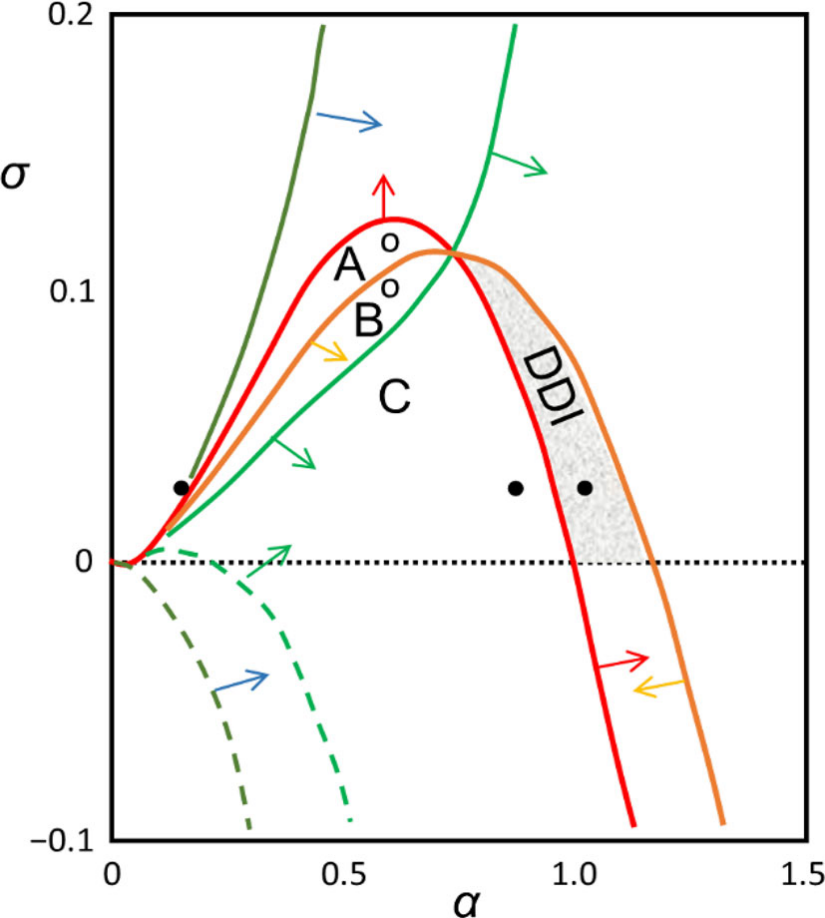
Region of diffusion-driven instability (DDI) of the HSS of the Tyson-Kauffman model in parameter space (*α*, *β*). In reference to Eq. (33), the curves correspond to conditions: (i) solid red, (ii) dashed dark green, (iii) solid orange, (iv) solid dark green, (v) dashed bright green, and (vi) solid bright green. The arrows indicate the side of each curve that satisfies the inequalities in Eq. (33). The curves are computed for *ν* = 0.125. Homogeneous limit cycle oscillations are expected in regions A, B and C, which correspond to the labeled regions in Fig. 4. The three black dots and two white circles denote the parameter values used for the simulations in Suppl. Figs. S3, S4 and S5

The equality in (50) traces the locus of Hopf bifurcation points in the (*α*, *σ*) parameter plane, the solid red line in Fig. 8. The other five loci in Fig. 8 are the lines (ii)-(vi) in Fig. 4, projected onto the (*α*, *σ*) parameter plane. Homogeneous oscillations are observed in the regions A, B and C; Turing patterns develop from small amplitude perturbations in the region (DDI) of diffusion-driven instability (see Suppl. Fig. S3A). In region C, we expect homogeneous oscillations to give way to a stable, time-independent Turing pattern (see Suppl. Fig. S3B). In regions A and B, we expect homogeneous oscillations and no stable Turing patterns, as confirmed in Suppl. Fig. S4.

In Suppl. Text 3, we describe two classic examples of activator-amplified negative feedback loops: the Gierer-Meinhardt (Gierer, 1972) model of tentacle formation in *Hydra* and the Lengyel-Epstein (1991) model of pattern formation in the chlorite-iodide-malonic acid reaction.

## Relation of Turing Patterns to Chemical Oscillations and Wave Propagation

We have seen that Turing’s requirements for diffusion-driven instability of the HSS of a pair of RDEs imply that the Jacobian matrix at the steady state take one of two possible sign patterns, Eq. (20): either an activator-amplified negative feedback loop or an inhibitor-amplified negative feedback loop. For two-component chemical reaction systems, these two sign patterns are also associated with limit cycle oscillations arising from Hopf bifurcations of the kinetic steady state (Novak, 2008). For this reason, spontaneous, homogeneous oscillations are also observed in most classical examples of chemical Turing patterns: e.g., the Brusselator, the Gierer-Meinhardt models (their simple-depletion model and their activator-inhibitor model), Schnakenberg’s model, the Tyson-Kauffman model and the Lengyel-Epstein model. Nonetheless, the association of Turing patterns with chemical oscillations is not absolute, as illustrated by our revised version of Turing’s second model (Suppl. Text 2), where Turing patterns develop from a bistable reaction system.

Two-component reaction–diffusion systems with the two sign patterns in Eq. (20) are also associated with traveling waves of chemical activation: solitary waves, target patterns, rotating spiral waves, and scroll waves; see (Tyson, 1988). These waves are observed in regions of parameter space, close to oscillations, where the HSS is stable but ‘excitable.’ Furthermore, traveling waves require that the diffusion constant of Y, the self-activating variable, is large compared to X, which is precisely opposite the requirement for Turing patterns. The Tyson-Kauffman model, for *σ* = 0.025 and *α* = 0.15, has a stable, excitable HSS and supports a solitary traveling wave of speed ~ 1.3 su/tu, for *ν* = 1, *μ* = 0.1, as shown in Suppl. Fig. S5A. As *μ* increases, the speed and amplitude of the wave decrease, until the wave disappears at *μ* ≈ 0.35.

Tyson (1991) showed that the Tyson-Kauffman model gives a decent account of mitotic oscillations in frog egg extracts, for quite different values of the parameters: *α* = 0.07, *σ* = 0.009, and *T*_su_ = 0.5 min. These parameter values give a traveling wave of speed 2.8 (Suppl. Fig. S5B). In this case, the self-activating variable is ‘M-phase Promoting Factor’ (MPF, a dimer of Cdk1 and cyclin B), with a diffusion constant in cytoplasm *D*_y _≈ 300 μm^2^/min. So, the model predicts waves of MPF activation propagating through frog egg cytoplasm at a speed of 2.8 × *S*_su_/*T*_su_ = 2.8√(*D*_y_/*T*_su_) ≈ 70 μm/min. This estimate agrees quite well with Chang and Ferrell (2013), who reported waves of MPF activation traveling at a speed of ~ 60 μm/min along a capillary tube filled with frog egg cytoplasm.

## Conclusion

In 1952, Alan Turing published an unprecedented paper proving—by mathematical reasoning and numerical simulations (using the novel, stored-program computing machine at Manchester University)—that some chemical reactions taking place in an unstirred vessel coupled spatially by molecular diffusion may develop stable, time-independent, spatially non-uniform distributions of chemical concentrations, provided the rate constants of the chemical reactions and of molecular diffusion satisfy certain precise mathematical conditions. Turing suggested that this mechanism of chemical pattern formation by the interactions of reaction kinetics and spatial diffusion might provide a basis for cellular pattern formation (‘morphogenesis’) in developing organisms. Turing’s paper is long and dense (mathematically and chemically), and far ahead of contemporary ideas in chemistry and biology. Not surprisingly, it was ignored for more than a decade, until his ideas were picked up by some physical chemists and mathematically inclined biologists. This paper reviews Turing’s work and its early revitalization by Prigogine and coworkers (Prigogine, 1967; Prigogine, 1968; Lefever 1968), Gierer and Meinhardt (1972), Segel and Jackson (1972), Lacalli and Harrison (1979), and others.

Since then, there has been a remarkable flowering of work on Turing’s ideas. Marcon and Sharpe (2012) review the applicability of Turing patterns to specific aspects of biological morphogenesis. Meron (2019) reviews their relevance to vegetation patterns in arid regions. Konow et al (2021) review the evidence for Turing patterns in non-living, chemical systems. Vittadello et al (2021) discuss the robustness of Turing patterns in light of their basic design principles. Generalizing on periodic Turing patterns, Champneys et al (2021) describe the formation of localized spikes and localized patterns in RDEs and apply the theory to the role of small G-protein networks in establishing cell polarity. Krause et al (2021) review modern developments in the theory of pattern formation in reaction-transport equations under a variety of circumstances.

A characteristic requirement for two-component reaction networks to exhibit diffusion-driven instability is that the diffusion constant of the self-amplifying variable be less than (often much less than) the diffusion constant of the feedback variable. This constraint is lifted in networks with a third, non-diffusing node that interacts with the self-amplifying variable; say, a buffering species like starch in the chlorite-iodide-malonic acid system, the cytoskeleton or membrane-bound receptors in cells, or the extracellular matrix (Klika, 2012; Korvasova, 2015; Lengyel, 1991; Marcon, 2016; Vittadello, 2021).

Several groups have extended Turing’s ideas to chemical reaction networks with three or more components. Using an automated linear stability analysis, Marcon et al (2016) screened all three-node networks (> 5000 distinctly different Jacobian matrices) with two diffusible nodes and one non-diffusible node, for diffusion-driven instability of the HSS. In contrast to two-node RDEs, in three-node systems there is no longer any constraint on the ratio of diffusion constants of the two diffusible species. Extending this study, Scholes et al (2019)) found that ~ 60% of three-node two-diffuser biochemical reaction networks are able to form Turing patterns. Following up on these studies, Tica et al (2024) engineered a three-node gene regulatory network in bacteria that forms stationary, periodic, concentric stripes of fluorescent protein expression in growing colonies.

My goal in this article has been to provide an introduction to Alan Turing’s remarkable 1952 paper on a chemical theory of biological morphogenesis. For different perspectives on Turing’s theory, including (notably) the experimental evidence for Turing patterns arising in biological and chemical settings, I refer readers to excellent review articles by Murray (1990), Meinhardt (2012), Ball (2015) Konow (2021), Murray (1989), Grindrod (1991) and Keener (2021).

## Supplementary Information

Below is the link to the electronic supplementary material.

Supplementary file1 (PDF 2081 KB)

## Footnotes

1 In their review article, Gmitro & Scriven briefly mentioned Turing’s paper in a broader context of spatial pattern formation in physicochemical systems operating far from equilibrium, such as Bénard convection cells in fluids and thermoelastic deformations of materials. Like Turing, they presented a general linear stability analysis of the HSS; unlike Turing, they did not provide any numerical examples of spontaneous pattern formation in RDEs. Prigogine and colleagues used one of Turing’s hypothetical chemical reaction systems to illustrate the global stability of the homogeneous equilibrium state in a closed, isolated chemical reactor and the potential for instability of the kinetic steady state in an open reactor subject to the continuous dissipation of free energy. For the most part, their analyses were quite abstract; only Lefever presented a numerical simulation of pattern formation in a modified version of Turing’s reaction mechanism (Lefever 1968). The Prigogine school referred to these self-organizing spatial patterns as ‘dissipative structures’ (Lefever 2018).

2 Lefever (2018) argues that ‘diffusion-driven instability’ is ‘sloppy language’ because it implies that chemical reaction rates (scalar numbers) are mechanistically coupled to diffusive fluxes (vector quantities) in violation of Curie’s principle. Although the two processes are not coupled directly, they are coupled indirectly because diffusive fluxes change local concentrations (i.e., reaction rates) and local differences in reaction rates induce concentration gradients (i.e., diffusive fluxes), as described by reaction–diffusion equations. With these caveats in mind, I stick with the language of ‘diffusion-driven instability of the HSS’ because it usefully describes how a chemical steady state that is stable with respect to local chemical reactions alone can be rendered unstable when local concentration changes induced by diffusive fluxes are taken into account.

3 Traditionally the two sign patterns are called an ‘activator-inhibitor model’ and a ‘substrate-depletion model,’ respectively (following Gierer and Meinhardt 1972), but these names are a little misleading because both options involve an activator-inhibitor NFL (because *bc* < 0). The fundamental distinction is whether it is the activator or the inhibitor of the NFL that is self-amplifying. In defense of the traditional terminology, one may think of the ‘self-amplifying’ species as the ‘activator’ and the ‘feedback’ species as either an ‘inhibitor’ or a ‘substrate,’ depending on which sign pattern (20) is under consideration. However, it is easy to imagine an inhibitor-amplified negative feedback loop where the feedback variable is not a ‘substrate’.

4 Turing was the first person to show, by numerical examples, that the partial differential equations describing the interaction of local chemical reactions and unbiased chemical diffusion may exhibit stable, time-independent, spatially nonuniform distributions of chemical concentrations, arising from a spatially homogeneous steady state (HSS) distribution that is unstable with respect to small perturbations of specific finite wavelength *λ*, 0 < *λ*_min_ < *λ* < *λ*_max_ < ∞. For this reason, it seems fitting to refer to such solutions as ‘Turing patterns.’ By this definition, Turing patterns are always associated with diffusion-driven instability of the HSS; however, as this example shows, stable Turing patterns often persist outside the DDI domain of Fig. 4. Furthermore, a HSS that is diffusively unstable does not necessarily develop into a persistent, stable Turing pattern, as shown by examples in Scholes et al (2019)—see their Fig. 3D (compare ‘Multistable Cases 1, 2’)—and Krause et al (2023)—their Fig. 1(a, e). In both examples, the reaction system has multiple steady states, with one HSS in the DDI domain and the other being stable with respect to all wavelength disturbances. A transient Turing pattern arises from the first unstable HSS but eventually gives ground to the other stable HSS.

5 Turing carries through a linear stability analysis of the HSS of Model 2 on p. 65. To understand it, one must be aware of Turing’s nomenclature. His *h* and *k* are our *X** and *Y**, respectively, i.e., *k* = 16 – *φ* > 0 and *h* = *α*/*k* = $\frac{16}{16-\varphi }>0$. Condition (28) implies $\frac{k}{4}+\frac{4}{k}=\frac{11}{4}$, or 6.725 < *φ* < 14.275, and condition (29) implies $\frac{k}{8}+\frac{8}{k}=\frac{5}{2}$ (note the misprint on p. 65), or 12 < *φ* < 16. Because Turing chooses *φ* = 12, his parameter set is right on the border of diffusion-driven instability of the HSS.
