# Correction: Revisiting Turing’s Chemical Basis of Morphogenesis

**DOI:** 10.1007/s11538-026-01657-9

**Published:** 2026-05-27

**Authors:** John J. Tyson

**Affiliations:** 1 Department of Biological Sciences, Virginia Polytechnic Institute and State University, Blacksburg, VA 24061 USA; 2 Present Address: 783 Spring Lane, Lansdale, PA 19446 USA

**Correction to: Bulletin of Mathematical Biology (2026) 88:76** 10.1007/s11538-026-01629-z

In this article, the formatting error was occurred during correction process due to production error and have now been corrected in the original publication. For completeness and transparency, the old incorrect versions are displayed below

1. Above Eq. (34), the lines should read as given below

As we saw HSS is X^*^ ≈ Y^*^ = 1, provided $\varphi = \alpha + \beta - 1$ and 0 < *ε* ≪ 1, and the elements of the Jacobian matrix are

Incorrect: 0 < ε]] > ≪ < ! [CDAT A[1

Corrected: 0 < *ε* ≪ 1

2. Eq. (40) should read as given below

Incorrect:

40 $$ J = \left[ {\begin{array}{*{20}c} a & b \\ c & d \\ \end{array} } \right]]] > \approx < ![CDATA\;A[\left[ {\begin{array}{*{20}c} { - \left[ {\alpha (\beta - 1) - \varphi } \right]} & { - \alpha /\left[ {\alpha (\beta - 1) - \varphi } \right]} \\ {(\beta - 1)\left[ {\alpha (\beta - 1) - \varphi } \right]} & {\varphi /\left[ {\alpha (\beta - 1) - \varphi } \right]} \\ \end{array} } \right] $$

Corrected:

40 $$ J = \left[ {\begin{array}{*{20}c} a & b \\ c & d \\ \end{array} } \right] \approx \left[ {\begin{array}{*{20}c} { - \left[ {\alpha \left( {\beta - 1} \right) - \varphi } \right]} & { - \alpha /\left[ {\alpha \left( {\beta - 1} \right) - \varphi } \right]} \\ {\left( {\beta - 1} \right)\left[ {\alpha \left( {\beta - 1} \right) - \varphi } \right]} & {\varphi /\left[ {\alpha \left( {\beta - 1} \right) - \varphi } \right]} \\ \end{array} } \right] . $$

3. Above Eq. (50), the line should be corrected as given below

Incorrect:

$$ {\mathrm{provided}}\;d > 0,\;{\mathrm{i}}.{\mathrm{e}}.,\;0 < \sigma < d^{{2}} ;\;{\mathrm{and}}\;a + d < 0,\;{\mathrm{i}}.{\mathrm{e}}.,\;0 < \sigma < {1}\;{\mathrm{and,}} $$

Corrected:

provided *d* > 0, i.e., 0 < *σ* < *α*^2^; and *a* + *d* < 0, i.e., 0 < *α* < 1 and

4. Below Eq. (41), the line should be corrected as given below

Incorrect:

 which is satisfied if $0<\varphi <\alpha \left(\beta -1\right)+1-\sqrt{1+\alpha \left(\beta -1\right)}<\alpha (\beta -1)$. For

Corrected:

which is satisfied if $0<\varphi <\alpha \left(\beta -1\right)-\frac{1}{2} \left(\sqrt{1+\alpha \left(\beta -1\right)}{-1} \right)<\alpha (\beta -1)$. For

And the following lines should be corrected as given below

Incorrect:

this condition implies that 0 < *φ* < 12.88.

Corrected:

 this condition implies that 0 < *φ* < 12.47.

