# Supplementary material for: Revisiting Turing’s Chemical Basis of Morphogenesis
Source: Bull Math Biol. 2026 Apr 7;88(5):76. doi: 10.1007/s11538-026-01629-z (PMC13056747; doi:10.1007/s11538-026-01629-z)
Supplement: Supplementary file 1 — Supplementary file1 (PDF 2081 KB) [file 11538_2026_1629_MOESM1_ESM.pdf]

Supplementary Information

Revisiting Turing's Chemical Basis of Morphogenesis

Bulletin of Mathematical Biology

John J. Tyson
Virginia Tech
tyson@vt.edu

© February 2026

Suppl. Code 1 MATLAB code for solving reaction-diffusion equations

Suppl. Table 1 Scaling factors for all models

Suppl. Fig. S1 Stable spatial patterns for Turing's first model for $v > ½$

Suppl. Fig. S2 Stable spatial patterns for Turing's second model

Suppl. Fig. S3 Stable spatial patterns for the Tyson-Kauffman model, Eqs. (49-50), in regions DDI and C of the Lacalli-Harrison diagram, Fig. 17

Suppl. Fig. S4 Simulations of the Tyson-Kauffman model, Eqs. (49-50), in regions A and B of the Lacalli-Harrison diagram, Fig. 17

Suppl. Fig. S5 Traveling wave for the Tyson-Kauffman model, Eqs. (49-50)

Suppl. Text 1 Revised version of Model 1

Suppl. Text 2 Revised versions of Model 2

Suppl. Text 3 Activator amplified negative feedback loops

Suppl. Code 1 MATLAB code for solving reaction-diffusion equations

The following code solves the RDEs for Turing's Model 1, using the MATLAB function pdepe (www.mathworks.com/help/matlab/ref/pdepe.html), and displays the solution as surface plots of $X(s,t)$ and $Y(s,t)$. The code is easily modified to solve all the other models described in the main text.

```
function pdeTuring1

%%%%%%%%%%%%%%%   Model parameters
mu = 1;
nu = 0.25;
alf = 3.5;
bet = 25;
phi = 27.5;
eps = 0.001;
qstar = 2.8;
L = 7;
TFinal = 100;

%%%%%%%%%%%%%%%   Define dimension, mesh and time span
m = 0;
x = linspace(0, L, 141);
t = linspace(0, TFinal, 25);

%%%%%%%%%%%%%%%      Solve PDE
sol = pdepe(m, @pdeTur1_pde, @pdeTur1_ic, @pdeTur1_bc, x, t);
u1 = sol(:,:,1);
u2 = sol(:,:,2);

%%%%%%%%%%%%%%%     Plot results
figure;
surf(x,t,u1)
title('X(s,t)')
xlabel('Distance s');
ylabel('Time t');

figure;
surf(x,t,u2)
title('Y(s,t)')
xlabel('Distance s');
ylabel('Time t');

%%%%%%%%%%%%%%%      PDE function
   function [c,f,s] = pdeTur1_pde(~,~,u,DuDx)
      c = [1; 1];
      f = [mu; nu] .* DuDx;
```

```
%%%%%%%%%%%%%%%      Reaction kinetics go here [ dx/dt; dy/dt ];
        s = [1-alf*u(1)*u(1)-bet*u(1)*u(2)+phi*u(2)/(eps+u(2));
             alf*u(1)*u(1)+bet*u(1)*u(2)-phi*u(2)/(eps+u(2))-u(2)];
    end

%%%%%%%%%%%%%%%%      Initial Conditions function
    function u0 = pdeTur1_ic(x)
         u0 = [1; 1+0.03*cos(qstar*x)];
    end

%%%%%%%%%%%%%%%%      Boundary Conditions function
    function [pl,ql,pr,qr] = pdeTur1_bc(~,~,~,~,~)
        pl = [0; 0];
        ql = [1; 1];
        pr = [0; 0];
        qr = [1; 1];
    end
end
```

Suppl. Table 1 Scaling factors for all models

Model	T_{su}	S_{su}	X_{su}	Y_{su}
1	$1/k_4$	$(D_x/k_4)^{1/2}$	k_1A/k_4	k_1A/k_4
1R	$1/k_4$	$(D_x/k_4)^{1/2}$	k_4/k_3	k_4/k_3
2	$1/k_4$	$(D_x/k_4)^{1/2}$	k_4K_d/k_2	k_4K_d/k_2
3	$1/k_6$	$(D_x/k_6)^{1/2}$	$(k_6K_d/k_7)^{1/2}$	$(k_6K_d/k_7)^{1/2}$
GM	$1/k_4$	$(D_x/k_4)^{1/2}$	$\frac{k_3}{k_1}\left(\frac{k_2K_{yx}}{k_4K_{yy}}\right)^2$	$\frac{k_2k_3}{k_1k_4}K_{yx}$
LE	$1/k_2B$	$(D/k_2B)^{1/2}$	L	k_2BL^2/k_3

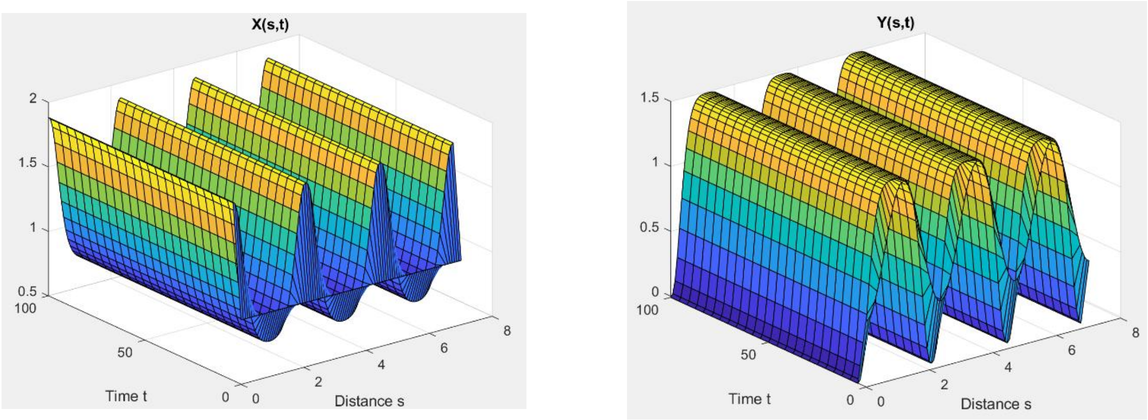

Suppl. Fig. S1 Stable spatial patterns for Turing's first model Eqs. (10-11) for $\nu > ½$. Parameter values: $\alpha = 3.5$, $\beta = 25$, $\varphi = \alpha + \beta - 1 = 27.5$, $\varepsilon = 0.001$, $\mu = 1$, $\nu = 0.64$. Initial conditions: $Y(s,0) = 1 - 0.5\cos(2\pi s/2.33)$, $X(s,0) = 1$. For $\nu > 0.65$, the system rapidly decays to the HSS (not shown)

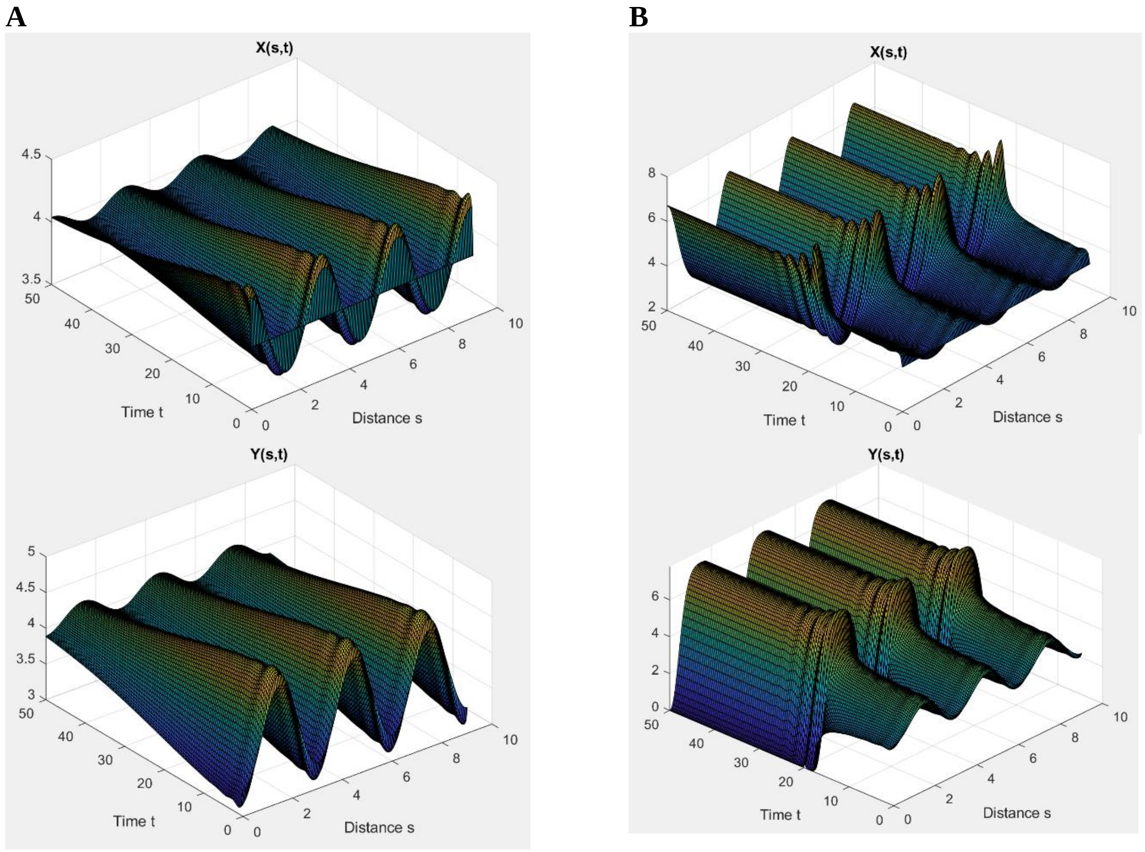

Suppl. Fig. S2 Stable spatial patterns for Turing's second model, Eqs. (37-38). Parameter values: $\alpha = 16$, $\beta = 2$, $\varphi = 12$, $\varepsilon = 0.012$, $\mu = 1$, $\nu = 0.25$; predicted $\lambda = 2\pi/\sqrt{Q_{\max}} = 3.14$. $L = 9$, $\Delta s = 9/180 = 0.05$. Initial conditions: $Y(s,0) = Y^*(1+A\cos(2\pi s/3))$, $X(s,0) = \alpha/Y^*$, with $Y^* \approx 4$. (**A**) $A = 0.16$. (**B**) $A = 0.18$; observed $\lambda = 3$

A **B**

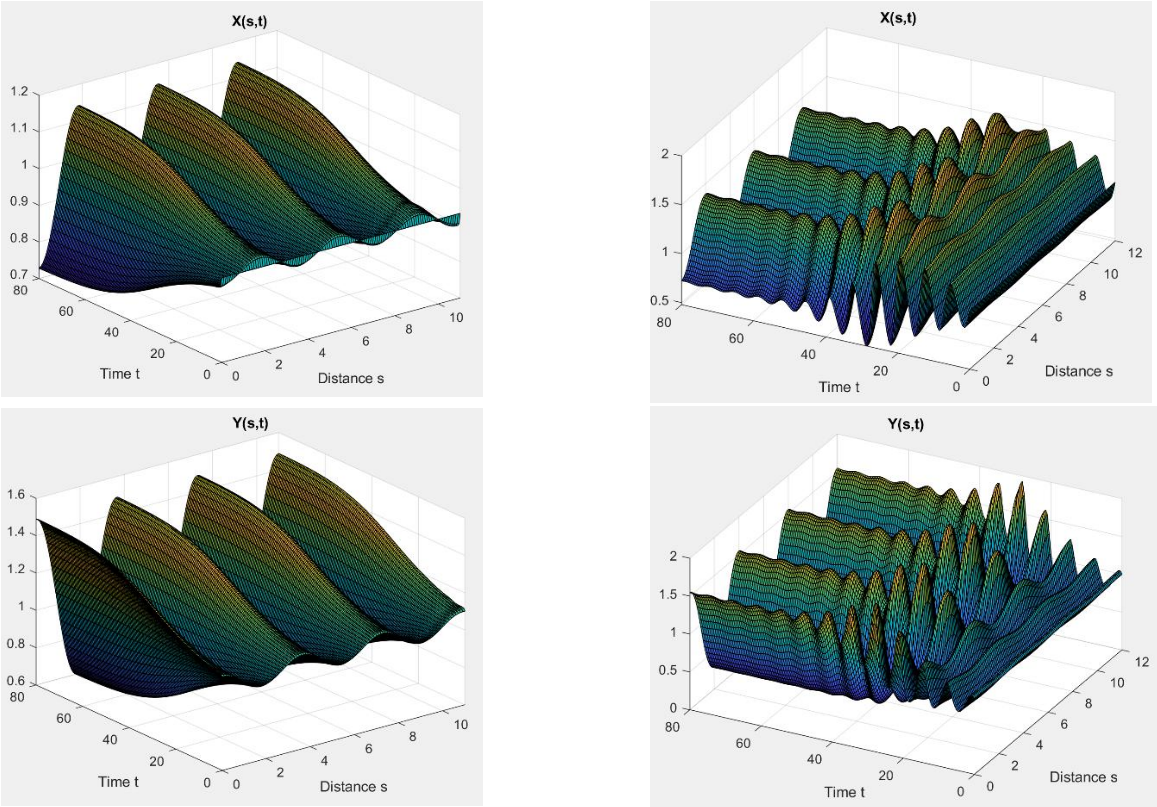

Suppl. Fig. S3 Stable spatial patterns for the Tyson-Kauffman model, Eqs. (45-46), with $\beta = \gamma = \delta = 0$, in regions DDI and C of the Lacalli-Harrison diagram, Fig. 8. Parameter values: $\sigma = 0.025$, $\mu = 1$, $\nu = 0.125$. (**A**) $\alpha = 1.05$. Turing pattern develops from a diffusive instability of the stable HSS. In this case, $a+d = -0.172$ and $ad-bc = 1.13$ (HSS is kinetically stable), $Q_{max} \approx 3$, $p_+(Q_{max}) \approx 0.05$, and $\lambda = 2\pi/\sqrt{Q_{max}} \approx 3.6$. Observed wavelength = 11/3 = 3.7. (**B**) $\alpha = 0.9$. Turing pattern develops from homogeneous oscillations around a kinetically unstable HSS. In this case, $a+d = 0.105$ and $ad-bc = 0.835$ (HSS is unstable, surrounded by limit cycle oscillations), $Q_{max} \approx 2.6$, $p_+(Q_{max}) \approx 0.165$, and $\lambda = 2\pi/\sqrt{Q_{max}} \approx 3.9$. Note: $p_+(Q_{max}) > (a+d)/2$, so the Turing pattern displaces the homogenous oscillation. For this simulation, $X(s,0) = X^* = 1.078$ and $Y(s,0) = 0.9\cdot(1.1 + 0.01\cdot\cos(1.61s)$. The large (10%) homogeneous perturbation excites homogeneous oscillations at first ($0 < t < 20$), which are then overtaken ($20 < t < 40$) by the stable Turing pattern excited by the small (1%) nonhomogeneous perturbation of wavelength ≈ 4. The Tyson-Kauffman model can be simulated in VisualPDE using the link https://visualpde.com/sim/?mini=KOM7tVY4

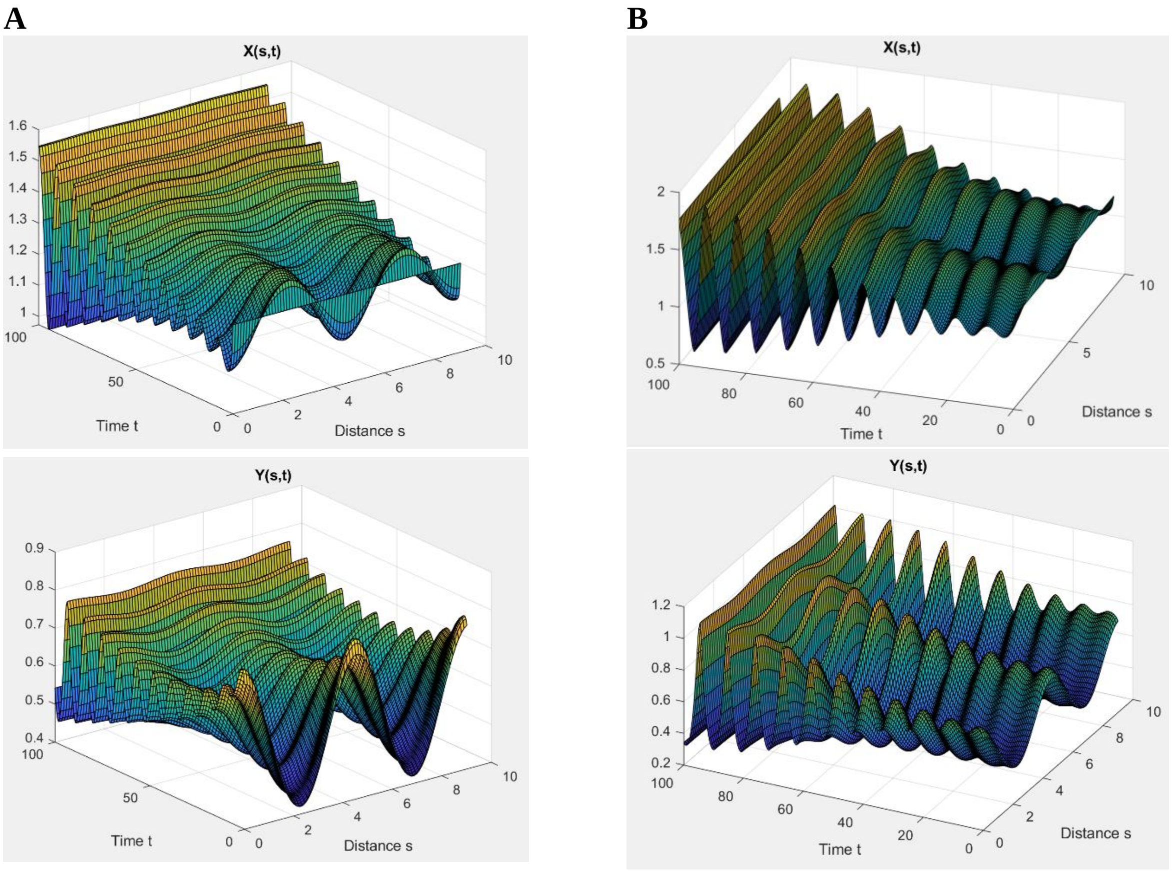

Suppl. Fig. S4 Simulations of the Tyson-Kauffman model, Eqs. (45-46), with $\beta = \gamma = \delta = 0$, in regions A and B of the Lacalli-Harrison diagram, Fig. 8. Parameter values: $\alpha = 0.6$, $\mu = 1$, $\nu = 0.125$. Initial conditions: $X(s,0) = X^*$, $Y(s,0) = Y^*(1 + 0.3\cos(q_{max}s))$. (**A**) Region A: $\sigma = 0.115$; $X^* = 1.26$, $Y^* = 0.6$; $q_{max} = 1.4$; $X(s,0) = X^*$, $Y(s,0) = Y^*\cdot(1 + 0.3\cos(q_{max}s))$. In this case, $(a+d)/2 = 0.018$ and $ad-bc = 0.475$ (HSS is unstable with respect to homogeneous limit cycle oscillations); $Q_{max} = 1.95$, $p_+(Q_{max}) = -0.028$. As expected, the Turing pattern of wavelength $= 2\pi/\sqrt{Q_{max}} \approx 4.5$ loses amplitude and the homogeneous oscillations grow in amplitude. (**B**) Region B: $\sigma = 0.1$; $X^* = 1.3$, $Y^* = 0.6$; $q_{max} = 1.38$; In this case, $(a+d)/2 = 0.053$ and $ad-bc = 0.46$ (HSS is unstable with respect to homogeneous limit cycle oscillations); $Q_{max} = 1.91$, $p_+(Q_{max}) = 0.026$ (HSS is unstable with respect to perturbations of wavelength $= 2\pi/\sqrt{Q_{max}} \approx 4.5$). The Turing pattern, which grows initially, is ultimately displaced by homogeneous limit cycle oscillations

A **B**

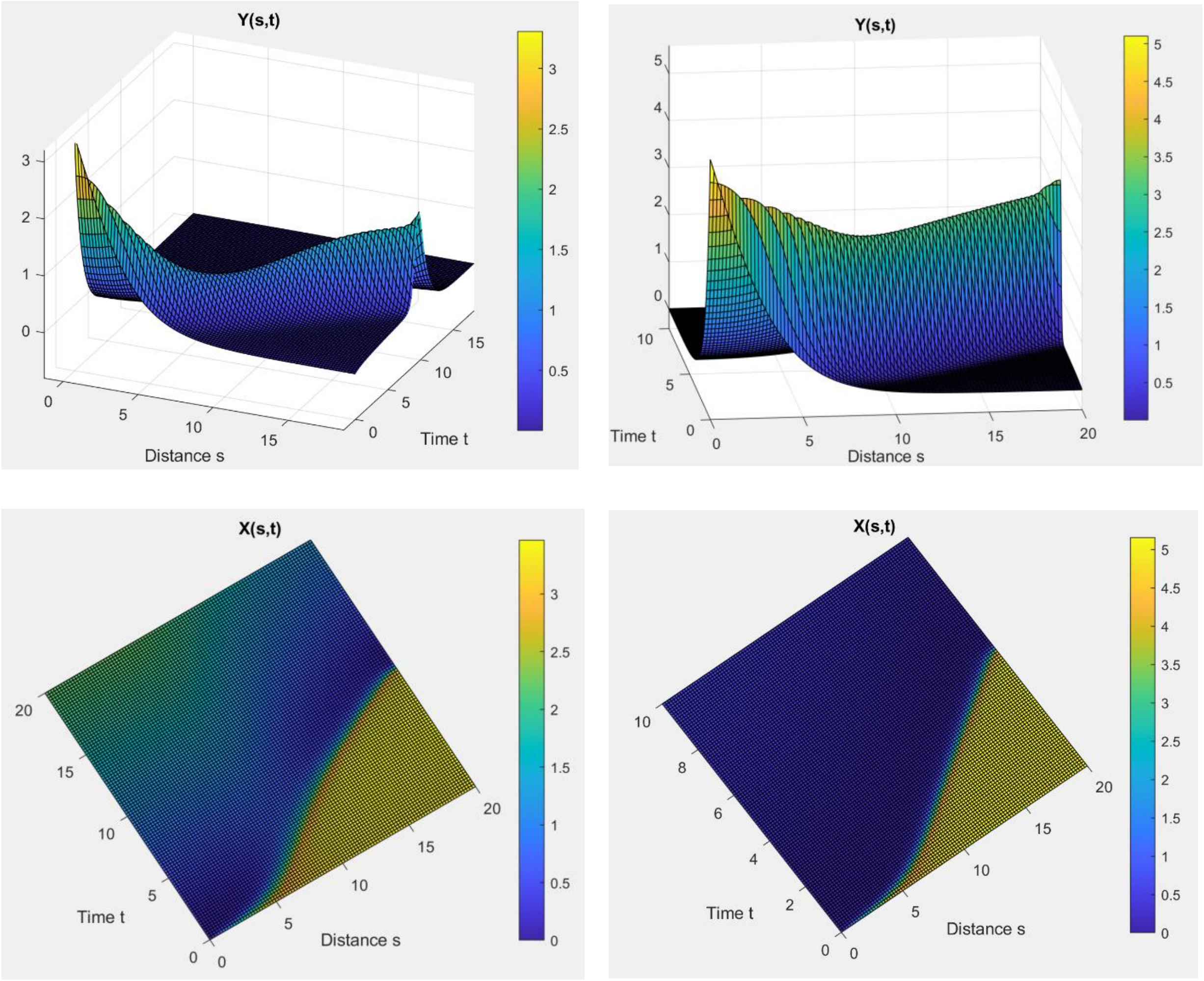

Suppl. Fig. S5 Traveling wave for the Tyson-Kauffman model, Eqs. (45-46), with $\beta = \gamma = \delta = 0$. (**A**) Parameter values: $\alpha = 0.15$, $\sigma = 0.025$, $\mu = 0.1$, $\nu = 1$; $X^* = 3.16$, $Y^* = 0.15$. Initial conditions: $X(s,0) = X^*+Y^* - Y(s,0)$, $Y(s,0) = 2(X^*+Y^*)/(1+\exp(s/2))$. A wave of activation of Y propagates along the tube, from $(s, t) = (0, 0)$ to $(L, 10)$, after which the medium returns to the HSS. Lower panel: this projection of $X(s,t)$ shows that the wave propagates across the middle of the tube ($8 < s < 12$) at a speed of ~1.3 su/tu. (**B**) Parameter values: $\alpha = 0.07$, $\sigma = 0.009$, $X^* = 5.036$, $Y^* = 0.07$; and same initial conditions as panel A. These parameter values are consistent with Fig. 3B of Tyson (1991). Note: $\mu = \nu = 1$ in this simulation, because active MPF (Y) and preMPF (X) have the same diffusion coefficients. Wave speed ≈ 2.8 su/tu

Suppl. Text 1 Revised version of Model 1

In his first model, Turing made a simplifying assumption, $\alpha + \beta = \varphi + 1$, so that the kinetic steady state is always at $X^* \approx 1$, $Y^* = 1$. Although this assumption simplifies the algebra of calculating the Jacobian matrix and carrying out stability analysis of the HSS, it constrains the model to exhibit only stable kinetic steady states ($a + d < 0$, $ad - bc > 0$). We can lift this constraint and simplify Model 1 in the process by replacing reaction R1.2 with

R1.2′ X → Y Rate = $k_2'X$ $k_2' = 25\times10^{-5}$ s^{-1}

In addition, we choose slightly different parameters for reaction R1.5: $k_5 = 0.17$ s^{-1}, $E_\mathrm{T} = 10^{-10}$M. In this case,

$$\frac{\partial \hat{X}}{\partial \hat{t}} = D_\mathrm{x}\frac{\partial^2 \hat{X}}{\partial \hat{s}^2} + k_1 A - k_2'\hat{X} - k_3\hat{X}\hat{Y} + k_5\frac{E_\mathrm{T}\cdot\hat{Y}}{E_\mathrm{T}+\hat{Y}}, \qquad (1.1)$$

$$\frac{\partial \hat{Y}}{\partial \hat{t}} = D_\mathrm{y}\frac{\partial^2 \hat{Y}}{\partial \hat{s}^2} + k_2'\hat{X} + k_3\hat{X}\hat{Y} - k_4\hat{Y} - k_5\frac{E_\mathrm{T}\cdot\hat{Y}}{E_\mathrm{T}+\hat{Y}}. \qquad (1.2)$$

Using the scaling factors in Suppl. Table 1, we cast these differential equations in dimensionless form

$$\frac{\partial X}{\partial t} = \mu\frac{\partial^2 X}{\partial s^2} + \alpha - \beta X - XY + \frac{\gamma Y}{\varepsilon + Y}, \qquad (1.3)$$

$$\frac{\partial Y}{\partial t} = \nu\frac{\partial^2 Y}{\partial s^2} + \beta X + XY - \frac{\gamma Y}{\varepsilon + Y} - Y, \qquad (1.4)$$

where

$$\alpha = \frac{k_3}{k_4}\cdot\frac{k_1 A}{k_4} = 25, \qquad \beta = \frac{k_2'}{k_4} = 4, \qquad \gamma = \frac{k_5}{k_4}\cdot\frac{k_3 E_\mathrm{T}}{k_4} = 680,$$
$$\varepsilon = \frac{k_3 E_\mathrm{T}}{k_4} = 0.25, \qquad \mu = 1, \qquad \nu = \frac{D_\mathrm{y}}{D_\mathrm{x}} = 0.5\,. \qquad (1.5)$$

The HSS solution of Eqs. (1.3-1.4) is $Y^* = \alpha$ and $X^* = \frac{\alpha + \alpha\gamma(\alpha+\varepsilon)^{-1}}{\alpha+\beta}$. In this case, the Jacobian matrix of the revised model is

$$\begin{bmatrix} a & b \\ c & d \end{bmatrix} = \begin{bmatrix} -(\alpha+\beta) & -X^* + \dfrac{\varepsilon\gamma}{(\alpha+\varepsilon)^2} \\ \alpha+\beta & X^* - \dfrac{\varepsilon\gamma}{(\alpha+\varepsilon)^2} - 1 \end{bmatrix} \qquad (1.6)$$

with determinant and trace given by

$$ad - bc = \alpha + \beta > 0\ ,\ \ a + d = -(\alpha + \beta) + \frac{\alpha + \alpha\gamma/(\alpha+\varepsilon)}{\alpha+\beta} - \frac{\varepsilon\gamma}{(\alpha+\varepsilon)^2} - 1 \qquad (1.7)$$

In this case, Model 1R exhibits Hopf bifurcations to spontaneous limit cycle oscillations when $a + d = 0$, as illustrated by the phase plane portrait in Suppl. Text 1 Fig. 1A. For the parameter values given in this figure ($\varepsilon = 0.25$, $\beta = 4$), the HSS is unstable ($a + d > 0$) and the system executes stable oscillations in the U-shaped region in the (α, γ) parameter space in panel B. The region of diffusion-driven instability (DDI) of the HSS and Turing patterns lies to the large-α side of the oscillatory region in panel B, as confirmed in Suppl. Text 1 Fig. 2.

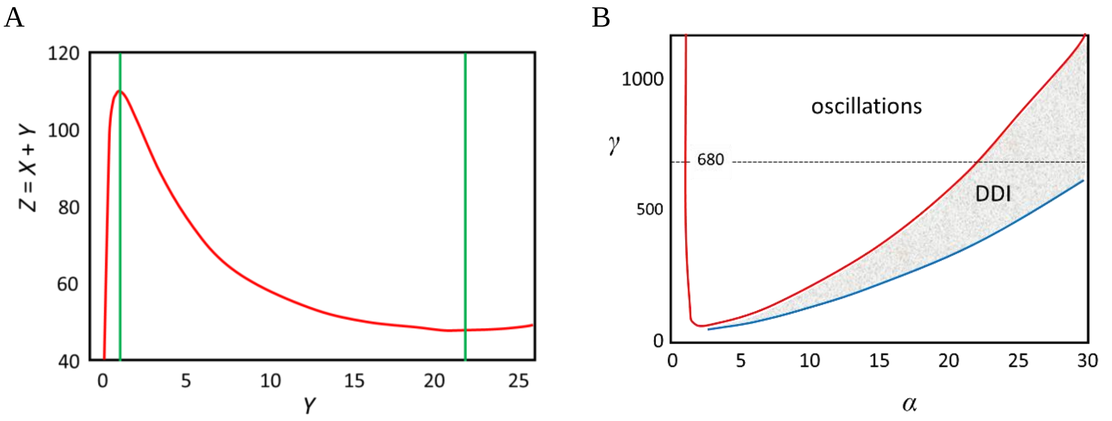

Suppl. Text 1 Fig. 1 Dynamic properties of Model 1R. (**A**) Phase plane ($Z = X + Y$ vs. Y). Red curve: Z nullcline for $\beta = 4$, $\varepsilon = 0.25$, $\gamma = 680$. Green curves: Y nullcline for $\alpha = 1.0345$ (left) and 21.67 (right). At these values of α, the steady state undergoes Hopf bifurcations to limit cycle oscillations. (**B**) Two-parameter bifurcation diagram (γ vs. α). Red curve: $a + d = 0$; blue curve: $d - \nu a = 2\sqrt{(-bc\nu)}$, for ν = 0.33. Homogeneous oscillations are observed in the region bounded by the red curve; Turing patterns arise from diffusion-driven instability of the HSS in the shaded region between the red and blue curves. Compare to the Lacalli-Harrison diagram in Fig. 4; there is no 'third side' to the DDI region because condition (ii) is $ad - bc > 0$, which is satisfied for all values of γ

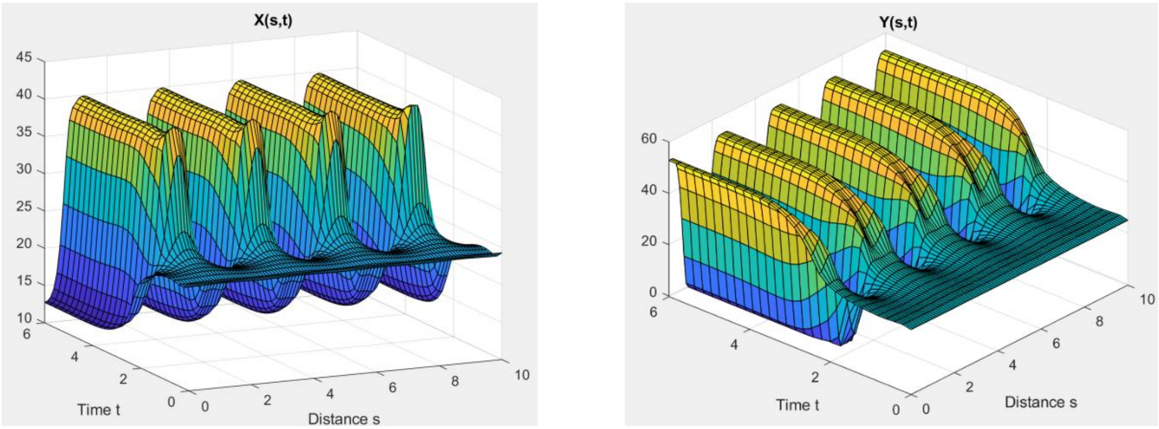

Suppl. Text 1 Fig. 2 Stable spatial pattern for Model 1R. Parameter values: $\alpha = 25$, $\beta = 4$, $\varepsilon = 0.25$, $\gamma = 680$, $\mu = 1$, $\nu = 0.33$. Initial conditions: $Y(s,0) = 25 + 0.025\cdot\cos(2\pi s/2.5)$, $X(s,0) = 24.31$

Suppl. Text 2 Revised versions of Model 2

Turing's second model has a major problem in that many trajectories 'blow up,' i.e., $Y(t) \to 0$ and $X(t) \to \infty$; see Suppl. Text 2 Fig. 1A,B. Here, we explore two different fixes to this problem.

2.1 Bistable Model 2 In the first case, we add a reaction (R2.6) that degrades X when Y is absent (so $X(t)$ doesn't $\to \infty$) and a reaction (R2.7) that produces Y from B at a slow, constant rate when [X:Y] = 0, so $Y(t)$ doesn't $\to 0$:

(R2.6)	X → D	Rate = k_6X	$k_6 = 1.25\times10^{-5}$ s^{-1}
(R2.7)	B → Y	Rate = k_7B	$k_7 = 0.625$ s^{-1}, $B = 10^{-6}$ M

The reaction-diffusion equations are

$$\frac{\partial X}{\partial t} = \mu\frac{\partial^2 X}{\partial s^2} + \alpha - XY - \delta X \tag{2.1}$$

$$\frac{\partial Y}{\partial t} = \nu\frac{\partial^2 Y}{\partial s^2} + \sigma + (\beta - 1)XY - \frac{\varphi Y}{\varepsilon + Y} - Y \tag{2.2}$$

where $\alpha = 16$, $\beta = 2.1$, $\varphi = 12$, $\varepsilon = 0.012$, $\delta = k_6/k_4 = 0.2$ and $\sigma = k_2k_7B/k_4^2K_d = 0.1$. (Note the slight change in the value of β.) This dynamical system has one or three real, positive HSS, as illustrated by the phase plane portrait (X vs. $Z = X{\cdot}Y$) in Suppl. Text 2 Fig. 1C,D and in the bifurcation diagrams in Suppl. Text 2 Fig. 1E,F. For $\beta = 2.1$, the Jacobian matrix at the lower steady state (at $X^* = 3.04$, $Y^* = 5.06$) is $[a, b; c, d] \approx [-5.26, -3.04; 5.56, 2.34]$, and the condition for diffusion-driven instability is $2.34 + 5.26\nu > 2\sqrt{16.9\nu}$, or $0 < \nu < 0.15$. The stable pattern for $\nu = 0.1$ is provided in Suppl. Text 2 Fig. 3A.

2.2 Oscillatory Model 2 In the second case, we add reaction (R2.8) for the uncatalyzed conversion of X into Y:

(R2.8)	X → Y	Rate = k_8X	$k_8 = 1.25\times10^{-5}$ s^{-1}

The reaction-diffusion equations are

$$\frac{\partial X}{\partial t} = \mu\frac{\partial^2 X}{\partial s^2} + \alpha - XY - \delta X \tag{2.3}$$

$$\frac{\partial Y}{\partial t} = \nu\frac{\partial^2 Y}{\partial s^2} + \delta X + (\beta - 1)XY - \frac{\varphi Y}{\varepsilon + Y} - Y \tag{2.4}$$

where $\delta = k_8/k_4 = 0.2$. (In addition, I change the value of k_5 to 0.0075 s^{-1}, so $\varepsilon = 0.1$ and $\varphi = 12$, as before.) Going back to $\beta = 2$, we plot the nullclines of system (2.3-4) in the (Y, Z) phase plane (Text Fig. 2.2A), where $Z = X + Y$, and we find that the kinetic steady state is unstable for $7.29 < \alpha < 14.75$. Over this interval the unstable steady state is surrounded by large amplitude, stable limit cycles (Text Fig. 2.2B).

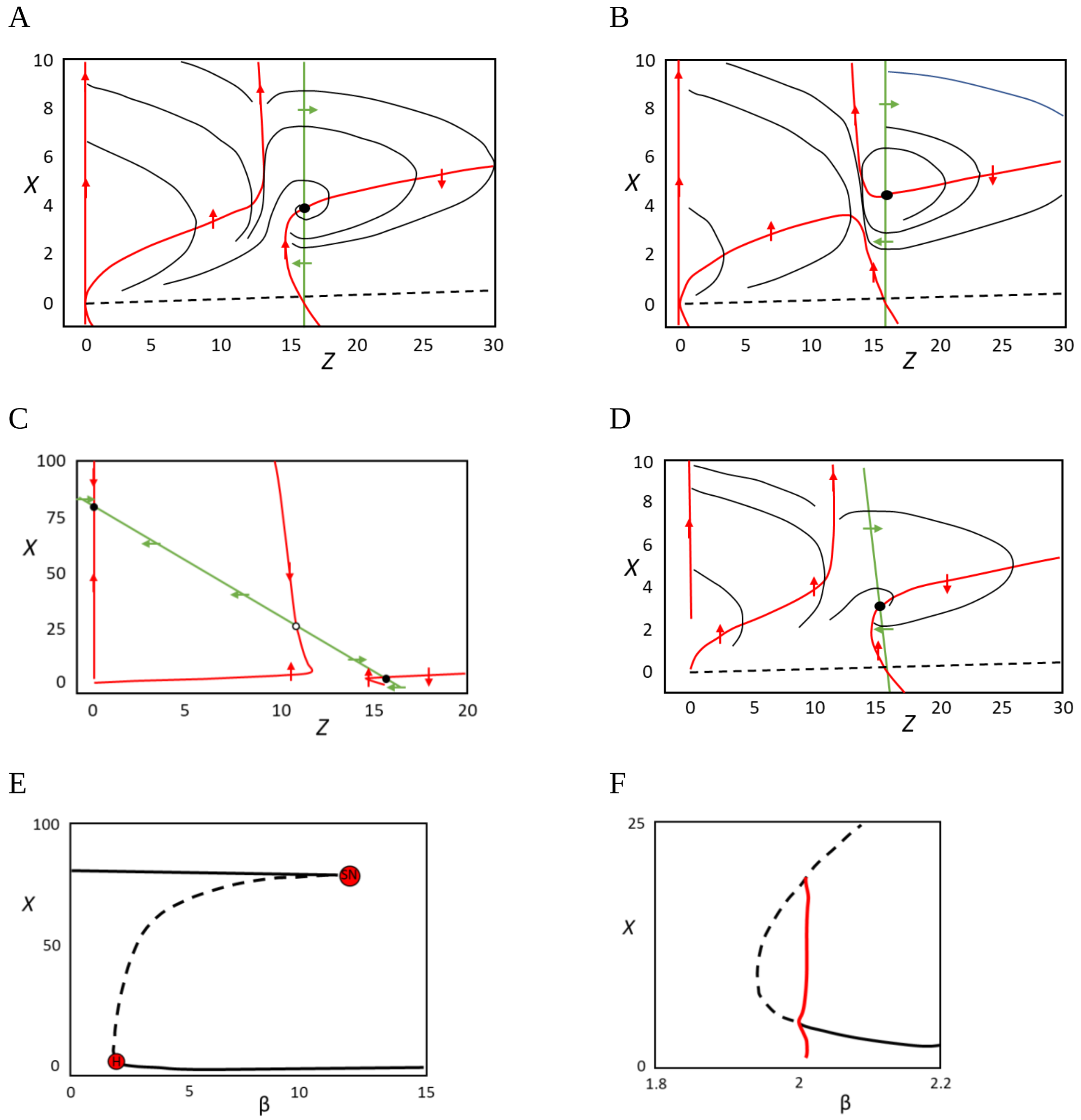

Suppl. Text 2 Fig. 1 Dynamics of Turing's second model. In all panels, $\alpha = 16$, $\varphi = 12$ and $\varepsilon = 0.012$. (**A, B**) Phase plane portraits (X vs. $Z = X{\cdot}Y$) for Turing's original Model 2, Eq. (37-38), for $\beta = 2$ and $\beta = 1.97$. The steady state at $Z = 16$, $X \approx 4$, is locally asymptotically stable, but large perturbations from the steady state blow up: $Z(t) \to 0$, $X(t) \to \infty$. Below the black dashed line, $Y > Z/X \approx 100$, is considered to be an 'unreachable' region of the phase plane. (**C, D**) Phase plane portraits (X vs. $Z = X{\cdot}Y$) for bistable Model 2R, Eq. (2.1-2.2), for $\beta = 2.1$, $\delta = 0.2$, $\sigma = 0.1$. Panel C illustrates the three steady states for this model at $(Z, X) \approx (15.4, 3)$, $(10.9, 25.5)$, $(0.009, 80)$. Panel D shows a close up of the lower stable steady state. (**E**) Bifurcation diagram (X vs. β) for bistable Model 2R. Solid lines: stable steady states (nodes); dashed lines: unstable steady states (saddle points). The lower steady state loses stability by a subcritical Hopf bifurcation (red circle) at $\beta = 2.007$. The upper stable steady state disappears at a saddle-node bifurcation at $\beta = 11.4$. The model is bistable for $2.01 < \beta < 11.4$. (**F**) Close-up of the subcritical Hopf bifurcation. The unstable limit cycles (red lines) are quickly extinguished at a saddle-loop bifurcation

To observe diffusion-driven instability of the HSS in this model, we must look outside this interval of limit cycle oscillations. For example, for $\alpha = 16$, the HSS at $X^* = 3.58$, $Y^* = 4.27$ is stable with a Jacobian matrix $[a, b; c, d] \approx [-4.47, -3.58; 4.47, 2.58]$, and the condition for diffusion-driven instability is $2.58 + 4.47\nu > 2\sqrt{16\nu}$, or $0 < \nu < 0.18$. The stable Turing pattern for $\nu = 0.1$ is provided in Text Fig. 2.3B.

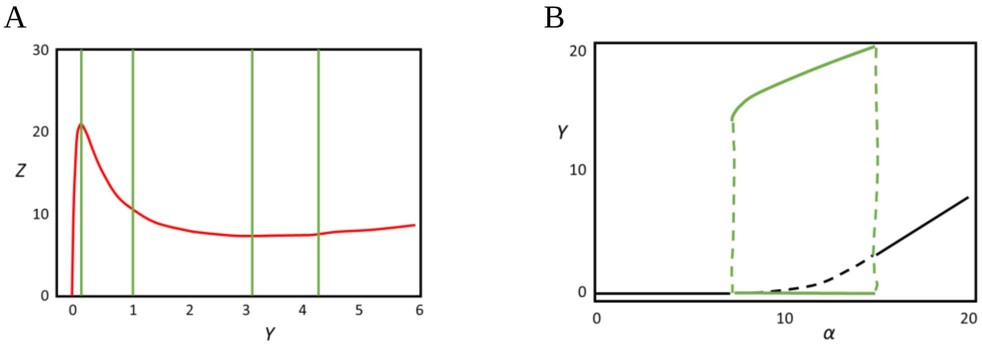

Suppl. Text 2 Fig. 2 Oscillatory Model 2R, Eqs. (2.3-3.4). Parameters $\beta = 2$, $\varphi = 12$, $\varepsilon = 0.1$, $\delta = 0.2$. (**A**) Phase plane portrait in the (Y vs. $Z = X+Y$) plane. Left-to-right: $\alpha = 7.29$, 12, 14.75, 16. (**B**) Bifurcation diagram, Y vs. α. The kinetic equations undergo subcritical Hopf bifurcations at $\alpha = 7.29$ and 14.75. Between these limits the system exhibits large amplitude, stable relaxation oscillations

A **B**

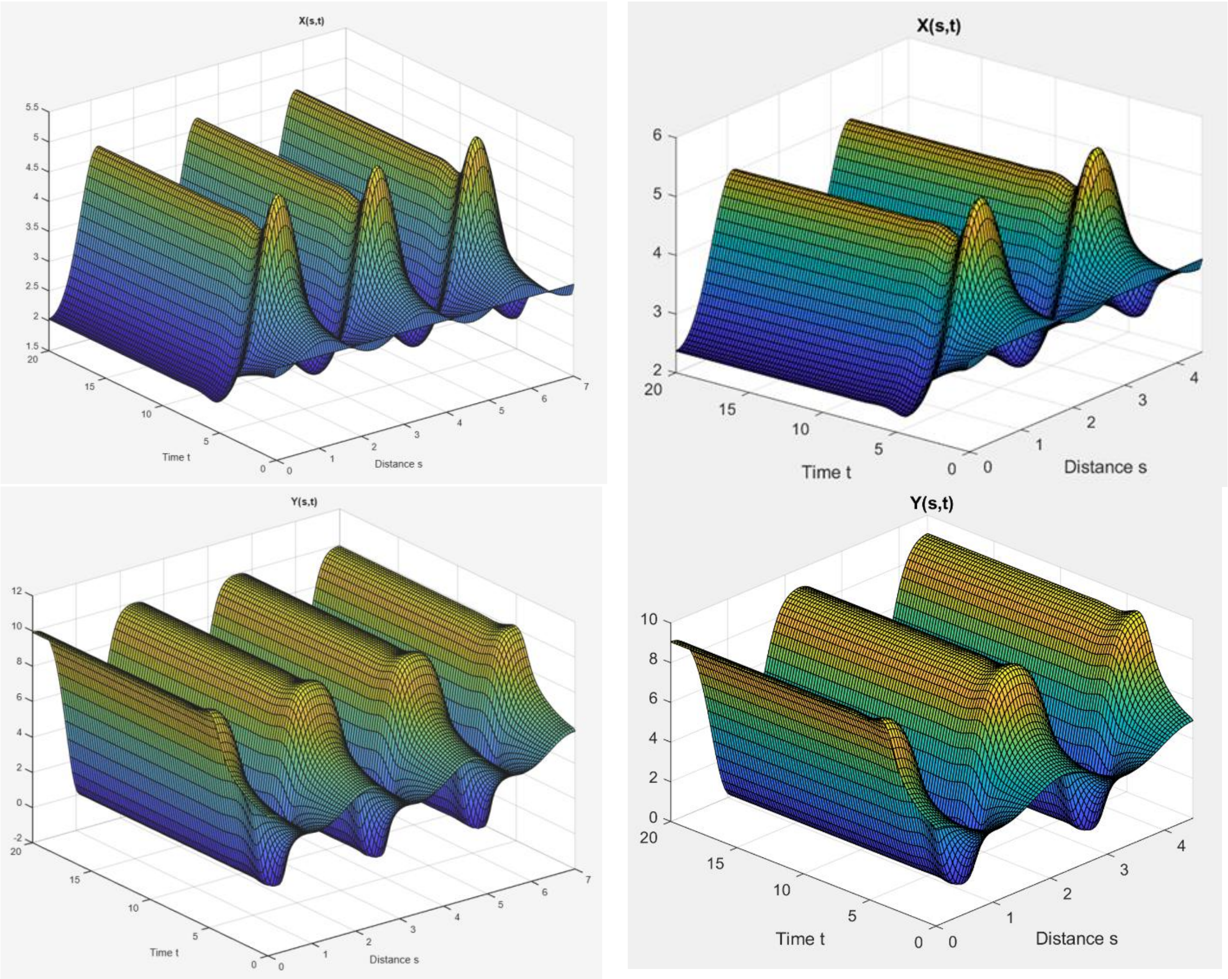

Suppl. Text 2 Fig. 3 Stable spatial patterns for the revised versions of Turing's second model. (**A**) Model 2R-Bi. Eqs. (2.1-2.2) Parameter values: α = 16, β = 2.1, φ = 12, δ = 0.1, σ = 0.1, ε = 0.012, μ = 1, ν = 0.1; in which case, $Q_{\max}$ = 7.5 and predicted $\lambda = 2\pi/\sqrt{Q_{\max}}$ = 2.3. L = 7, Δs = 7/140 = 0.05. Initial conditions: $Y(s,0) = 5.06(1+0.15\cos(2\pi s/2.33))$, $X(s,0)$ = 3.04. (**B**) Model 2R-Os, Eqs. (2.3-2.4). Parameter values: α = 16, β = 2, φ = 12, δ = 0.2, ε = 0.1, μ = 1, ν = 0.1; in which case, $Q_{\max}$ = 7.63 and predicted $\lambda = 2\pi/\sqrt{Q_{\max}}$ = 2.27. L = 4.5, Δs = 0.05. Initial conditions: $Y(s,0)$ = 4.27(1+0.15cos($2\pi s$/2.25)), $X(s,0)$ = 3.58

Suppl. Text 3 Activator-amplified negative feedback loops

Models 1-3 are all based on an inhibitor-amplified negative feedback loop with a Jacobian matrix of sign pattern $\begin{bmatrix} - & - \\ + & + \end{bmatrix}$. Diffusion-driven instabilities are also possible for activator-amplified negative feedback loops (AANFL) with sign pattern $\begin{bmatrix} - & + \\ - & + \end{bmatrix}$. In 1972 Gierer & Meinhardt introduced the first example of Turing patterns in a hypothetical AANFL based on gene regulation. In 1979 Lengyel & Epstein proposed an AANFL model of Turing patterns in the Chlorite-Iodide-Malonic Acid-Starch reaction, based on a mechanism whereby iodide ions inhibit their own removal by reacting with chlorite ions ($ClO_2^- + 4I^- + 4H^+ \rightarrow Cl^- + 2I_2 + 2H_2O$). In this supplement, we examine these informative models in some detail.

3.1 Gierer & Meinhardt's 'activator-inhibitor' model A simplified version of the Geirer-Meinhardt (1972) model is presented in Suppl. Text 3 Fig. 1 and Suppl. Text 3 Table 1.

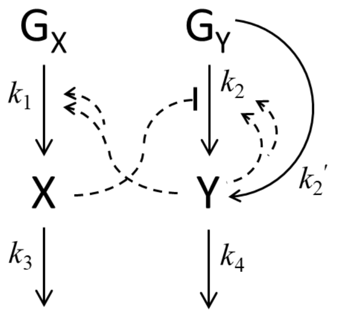

Suppl. Text 3 Fig. 1 Gierer-Meinhardt model. Proteins X and Y control the expression of genes G_X and G_Y. The transcription of both G_X and G_Y is activated by binding of two molecules of Y to upstream regulatory sequences. G_Y transcription is inhibited by binding of one molecule of X. Furthermore, there is a slow rate (k_2') of constitutive G_Y transcription

Suppl. Text 3 Table 1 Gierer-Meinhardt model*

R4.1	$G_X{:}Y_2 \rightarrow G_X{:}Y_2 + X$	Rate = $k_1 P(G_X{:}Y_2)$	$k_1 = 200$ nM min^{-1}
R4.2	$G_Y{:}Y_2 \rightarrow G_Y{:}Y_2 + Y$	Rate = $k_2 P(G_Y{:}Y_2)$	$k_2 = 100$ nM min^{-1}
R4.2′	$G_Y \rightarrow G_Y + Y$	Rate = k_2'	$k_2' = 0.05$ nM min^{-1}
R4.3	$X \rightarrow$	Rate = $k_3 X$	$k_3 = 0.01$ min^{-1}
R4.4	$Y \rightarrow$	Rate = $k_4 Y$	$k_4 = 0.01$ min^{-1}
R4.5	$G_X + 2Y \rightarrow G_X{:}Y_2$	$[G_X{:}Y_2] = [G_X](Y/K_{xy})^2$	$K_{xy} = 100$ nM
R4.6	$G_Y + 2Y \rightarrow G_Y{:}Y_2$	$[G_Y{:}Y_2] = [G_Y](Y/K_{yy})^2$	$K_{yy} = 100$ nM
R4.7	$G_Y + X \rightarrow X{:}G_Y$	$[X{:}G_Y] = [G_Y](X/K_{yx})$	$K_{yx} = 20$ nM
R4.8	$G_Y{:}Y_2 + X \rightarrow X{:}G_Y{:}Y_2$	$[X{:}G_Y{:}Y_2] = [G_Y{:}Y_2](X/K_{yx})$	

* We assume that $D_x = 200\ \mu m^2\ min^{-1}$ and $D_y = 3\ \mu m^2\ min^{-1}$.

The probabilities that genes G_X and G_Y are in the 'activated' state are

$$P(\mathrm{G_X}:\mathrm{Y_2}) = \frac{\hat{Y}^2}{K_{\mathrm{xy}}^2 + \hat{Y}^2}\,, \qquad P(\mathrm{G_Y}:\mathrm{Y_2}) = \frac{\hat{Y}^2}{K_{\mathrm{yy}}^2 + \hat{Y}^2} \cdot \frac{K_{\mathrm{yx}}}{K_{\mathrm{yx}} + \hat{X}}\,. \tag{3.1}$$

Hence, the RDEs for this model are

$$\frac{\partial \hat{X}}{\partial \hat{t}} = D_{\mathrm{x}} \frac{\partial^2 \hat{X}}{\partial \hat{s}^2} + k_1 \frac{\hat{Y}^2}{K_{\mathrm{xy}}^2 + \hat{Y}^2} - k_3 \hat{X}\,, \tag{3.2}$$

$$\frac{\partial \hat{Y}}{\partial \hat{t}} = D_{\mathrm{y}} \frac{\partial^2 \hat{Y}}{\partial \hat{s}^2} + k_2' + k_2 \frac{\hat{Y}^2}{K_{\mathrm{yy}}^2 + \hat{Y}^2} \cdot \frac{K_{\mathrm{yx}}}{K_{\mathrm{yx}} + \hat{X}} - k_4 \hat{Y}\,. \tag{3.3}$$

(Note: to simplify the model, I am assuming that $K_{xy} = K_{yy}$, i.e., that Y binds equally well to the regulatory sequences of G_X and G_Y.) Using the standard units in Suppl. Table 1, we cast the model into dimensionless form

$$\frac{\partial X}{\partial t} = \mu \frac{\partial^2 X}{\partial s^2} + \kappa \left[\frac{Y^2}{1 + \varepsilon^2 Y^2} - X \right], \tag{3.4}$$

$$\frac{\partial Y}{\partial t} = \nu \frac{\partial^2 Y}{\partial s^2} + \alpha + \frac{Y^2}{1 + \varepsilon^2 Y^2} \cdot \frac{1}{\theta + X} - Y\,, \tag{3.5}$$

where the dimensionless parameters are

$$\varepsilon = \frac{k_2 k_3 K_{\mathrm{yx}}}{k_1 k_4 K_{\mathrm{yy}}} = 0.1, \qquad \kappa = \frac{k_3}{k_4} = 1,$$
$$\alpha = \frac{k_1 k_2'}{k_2 k_3 K_{\mathrm{yx}}} = 0.5, \qquad \theta = \frac{k_1 k_4^2 K_{\mathrm{yy}}^2}{k_3 k_2^2 K_{\mathrm{yx}}} = 0.1, \tag{3.6}$$
$$\mu = 1, \qquad \nu = \frac{D_{\mathrm{y}}}{D_{\mathrm{x}}} = 0.015.$$

To simplify the algebra, let's assume (for the time being) that $\varepsilon Y \ll 1$ and $\theta \ll X$. In this case, the steady state solution of Eqs. (3.4-5) is $Y^* \approx 1+\alpha = 1.5$ (which is smallish compared to ε^{-1}) and $X^* = (1+\alpha)^2 = 2.25$ (which is large compared to θ). The Jacobian matrix for the Gierer-Meinhardt model is

$$J = \begin{bmatrix} a & b \\ c & d \end{bmatrix} \approx \begin{bmatrix} -\kappa & 2\kappa(1+\alpha) \\ \dfrac{-1}{(1+\alpha)^2} & \dfrac{1-\alpha}{1+\alpha} \end{bmatrix}. \tag{3.7}$$

The determinant and trace of this matrix are

$$ad - bc = \kappa, \text{ and } \quad a + d = -\kappa + \frac{1-\alpha}{1+\alpha}\ . \qquad (3.8)$$

The conditions for diffusion-driven instability are $0 < \alpha < 1$, $\kappa > \frac{1-\alpha}{1+\alpha}$, and $\kappa\nu - \sqrt{\frac{8\kappa\nu}{1+\alpha}} + \frac{1-\alpha}{1+\alpha} > 0$. The last inequality demands that

$$0 < \nu < \frac{1}{\kappa}\left(\sqrt{\frac{2}{1+\alpha}} - 1\right)^2 . \qquad (3.9)$$

For $\alpha = 0.5$ and $\kappa = 1$, this condition implies that $0 < \nu < 0.024$, which is satisfied for our choice of $D_y/D_x = 0.015$. In this case, $Q_{max} \approx 8.36$, or an expected wavelength $\approx 2\pi/2.9 = 2.17 = 300$ µm. The Turing pattern that develops from Eqs. (3.4-5), with the parameter values in Eq. (3.6), is illustrated in Suppl. Text 3 Fig. 2.

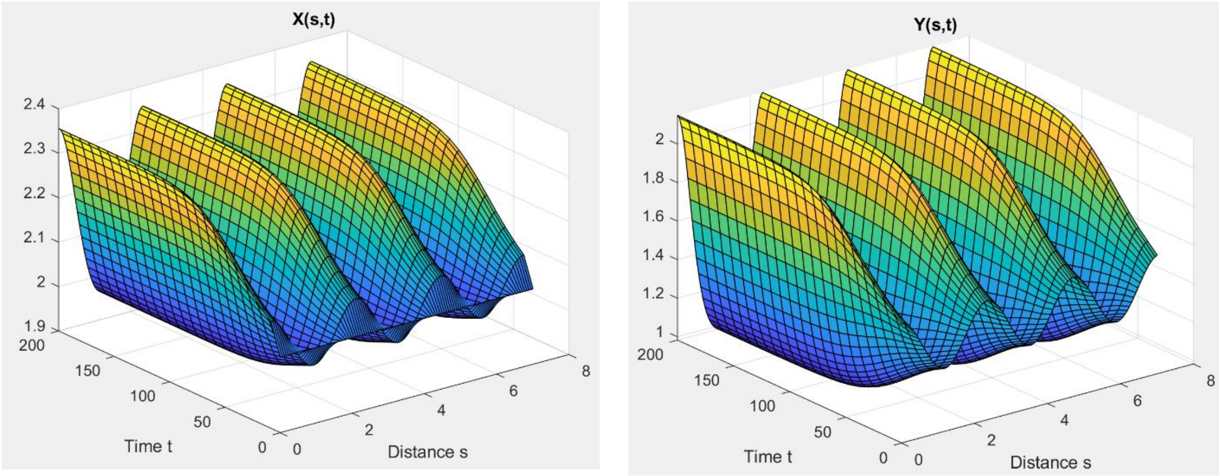

Suppl. Text 3 Fig. 2 Gierer-Meinhardt model, Eqs. (3.4-5), for the parameter values in Eq. (3.6)

3.2 Lengyel-Epstein model of a chemical Turing pattern The first unequivocal demonstration of a Turing pattern arising from the interaction of chemical reactions and diffusion was provided by Castets et al (1990) in a gel reactor loaded with starch and supplied with malonic acid on one side and a mixture of chlorite (ClO_2^-) and iodide (I^-) on the other (known as the 'CIMA' reaction). In the middle, there developed a pattern of dots regularly spaced at 200 µm intervals. Shortly thereafter, Lengyel & Epstein (1991) published a simple and elegant model of these structures, based on the underlying chemistry of the reaction mixture; see Suppl. Text 3 Fig. 3 and Suppl. Text 3 Table 2.

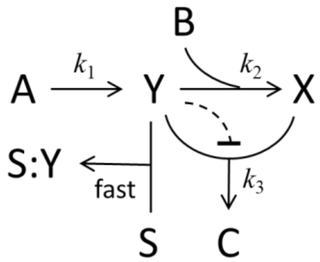

Suppl. Text 3 Fig. 3 Lengyel-Epstein model of the CIMA reaction. A = MA + I_2, Y = I^-, X = ClO_2^-, B = Cl_2 and S = starch

Suppl. Text 3 Table 2 Lengyel-Epstein model of the CIMA reaction*

R5.1	A → Y	Rate = k_1A	$k_1 = 10^{-3}$ s^{-1}, $A = 10^{-2}$ M
R5.2	B + Y → X	Rate = k_2BY	$k_2 = 10^3$ M^{-1}s^{-1}, $B = 10^{-2}$ M
R5.3	X + 4Y → C	Rate = $k_3\frac{XY}{L^2+Y^2}$	$k_3 = 1.5\times10^{-7}$ M s^{-1}, $L = 10^{-7}$ M
R5.4	S + Y → S:Y	pre-equil	[S:Y] ≈ S_TY/K_d, $K \equiv S_T/K_d = 49$

* Step 3 is a non-elementary reaction, inhibited by Y. For these small ions, $D = 1.5\times10^{-5}$ cm^2/s

Let $\hat{Y}_T = \hat{Y} + [S{:}Y] = (1+K)\hat{Y} = \frac{1}{\nu}\hat{Y}$, where ν = 1/(1+$K$) = 0.02. Then the RDE for 'free' Y is $\frac{1}{\nu}\frac{\partial\hat{Y}}{\partial\hat{t}} = \frac{\partial\hat{Y}_T}{\partial\hat{t}} = \frac{\partial\hat{Y}}{\partial\hat{t}} + \frac{\partial[S{:}Y]}{\partial\hat{t}}$. Hence, the RDEs for the Lengyel Epstein model are

$$\frac{\partial\hat{X}}{\partial\hat{t}} = D\frac{\partial^2\hat{Y}}{\partial\hat{s}^2} + k_2B\hat{Y} - k_3\frac{\hat{X}\hat{Y}}{L^2+\hat{Y}^2}, \tag{3.10}$$

$$\frac{\partial\hat{Y}}{\partial\hat{t}} = \nu D\frac{\partial^2\hat{Y}}{\partial\hat{s}^2} + \nu\left(k_1A - k_2B\hat{Y} - 4k_3\frac{\hat{X}\hat{Y}}{L^2+\hat{Y}^2}\right). \tag{3.11}$$

Notice that iodide binding to starch decreases both the effective diffusion constant of I^- and the rate of change of [I^-] compared to [ClO_2^-]. Note also the stoichiometric constant, 4, in the RDE for I^-. As before, we use the standard units in Suppl. Table 1 to cast Eqs. (3.10-11) into dimensionless form

$$\frac{\partial X}{\partial t} = \frac{\partial^2X}{\partial s^2} + \beta\left(Y - \frac{XY}{1+Y^2}\right), \tag{3.12}$$

$$\frac{\partial Y}{\partial t} = \nu\frac{\partial^2Y}{\partial s^2} + \nu\left(\alpha - Y - 4\frac{XY}{1+Y^2}\right), \tag{3.13}$$

where the dimensionless parameters are

$$\alpha = \frac{k_1 A}{k_2 BL} = 10, \qquad \beta = \frac{k_3}{k_2 BL} = 0.15, \qquad \nu = \frac{1}{1+K} = 0.02. \tag{3.14}$$

The steady state solution of this model is $Y^* = \alpha/5$ and $X^* = 1 + \alpha^2/25$, and the Jacobian matrix is

$$J = \begin{bmatrix} a & b \\ c & d \end{bmatrix} = \begin{bmatrix} \dfrac{-5\alpha\beta}{\alpha^2+25} & \dfrac{2\alpha^2\beta}{\alpha^2+25} \\ \dfrac{-20\alpha\nu}{\alpha^2+25} & \nu\dfrac{3\alpha^2-125}{\alpha^2+25} \end{bmatrix}. \tag{3.15}$$

The determinant and trace of this matrix are

$$ad - bc = \frac{25\alpha\beta\nu}{\alpha^2+25} > 0, \text{ and } \quad a + d = -\frac{5\alpha\beta - \nu(3\alpha^2-125)}{\alpha^2+25}\ . \tag{3.16}$$

The conditions for diffusion-driven instability are $\alpha > 6.455$, $\nu < \frac{5\alpha\beta}{\alpha^2+25}$, and, from Eq. (29) of the main text,

$$5\alpha\beta - 4\alpha\sqrt{10\alpha\beta} + (3\alpha^2 - 125) > 0\ . \tag{3.17}$$

The latter condition implies that $0 < \beta < \left(4\sqrt{\alpha/10} - \sqrt{(\alpha^2+25)/\alpha}\right)^2$, which, for $\alpha = 10$, implies that $0 < \beta < 0.216$. Our estimates, $\beta = 0.15$, $\nu = 0.02$, satisfy all these inequalities. In this case, the Jacobian matrix is $J = \begin{bmatrix} -0.06 & 0.24 \\ -0.032 & 0.028 \end{bmatrix}$. Hence, $Q_{max} \approx 0.5$, for an expected wavelength ≈ 2π/0.7 = 9 su ≈ 0.1 mm, which is not too far off from the observed wavelength of 0.2 mm.

Notice that the constraint on ν, the effective diffusion constant of free iodide ions, is determined, not by the usual constraint (29) of the main text, but by the condition $a + d < 0$, i.e., that the HSS be stable with respect to homogeneous perturbations.
